# Effectiveness of dual-task exercise in improving balance and preventing falls among older adults: systematic review with meta-analysis and meta-regression

**DOI:** 10.1007/s41999-025-01328-3

**Published:** 2025-10-28

**Authors:** Mohammad Jobair Khan, Kenneth N. K. Fong, Thomson Wai-Lung Wong, William Wai-nam Tsang, Cynthia Chen, Wai-chi Chan, Stanley John Winser

**Affiliations:** 1 Department of Rehabilitation Sciences, The Hong Kong Polytechnic University, Hong Kong, SAR China; 2 Department of Physiotherapy, Hong Kong Metropolitan University, Hong Kong, SAR China; 3 Saw Swee Hock School of Public Health, National University of Singapore, Singapore, Singapore; 4 Department of Psychiatry, The University of Hong Kong, Hong Kong, SAR China

**Keywords:** Elderly, Posture control, Accidental fall, Meta-regression, Motor–cognitive task

## Abstract

**Purpose:**

To investigate whether dual-task (DT) training improves dynamic balance and functional mobility and reduces falls and determine how DT training dosage, including exercise duration, intensity, challenge and adherence, affects treatment outcomes in older adults.

**Methods:**

Databases were searched until May 2025. Methodology, risk of bias and evidence quality were assessed using the Physiotherapy Evidence Database (PEDro), Cochrane risk of bias and Grading of Recommendations, Assessment, Development, and Evaluation tools, respectively. Dynamic balance was assessed using the Berg Balance Scale (BBS) and Fullerton Advanced Balance (FAB), and functional mobility using Timed Up and Go (TUG), and falls assessed the frequency of fall.

**Results:**

Forty-four studies involving 2782 older adults were included. The mean PEDro score was 6.1, 58% of studies were found to have low risk of bias, and evidence quality ranged from very low to moderate. Meta-analysis indicated that DT exercise significantly improves dynamic balance and functional mobility, and reduces fall frequency. Meta-regression revealed that dynamic balance improved with 95% adherence to 30 min of moderately challenging and moderate-intensity DT training three times per week for four weeks. Functional mobility improved with 95% adherence to 50 min of mildly challenging and moderate-intensity DT training three times per week for 13 weeks.

**Conclusion:**

DT training improved dynamic balance and functional mobility and reduced falls. The recommended dosages may elicit optimal outcomes. Additional meta-regression analyses are needed to determine the impact of DT training dosage on falls.

**Supplementary Information:**

The online version contains supplementary material available at 10.1007/s41999-025-01328-3.

## Key summary points

**Aim:**

To identify the optimal dosage of dual-task intervention required for improving dynamic balance and functional mobility and reducing falls in healthy older adults.

**Findings:**

Dynamic balance and functional mobility improved with 95% adherence to the prescribed dual-task training, practiced three times weekly. More studies are required to assess the dosage of dual-task training for fall reduction.

**Message:**

An adequate dosage of dual-task training requires improving dynamic balance and functional mobility.

**Supplementary Information:**

The online version contains supplementary material available at 10.1007/s41999-025-01328-3.

## Introduction

Each year, nearly one in four people over 65 years of age experiences one or more falls in the USA [1]. Falls lead to significant morbidity and mortality and substantially increase global healthcare costs [2]. Fall prevention in the ageing population is therefore a pressing public health issue [3]. Over a decade ago, national healthcare organisations in Australia and the UK actively tailored fall prevention training to older adults [4, 5]. However, a recent review revealed that many nations are far from adopting fall prevention programmes into their national healthcare agendas [6].

Although the causes of falls are multifaceted, impaired balance and cognition are significant contributing factors [7]. Extensive research has thus examined the interaction between balance and cognition following dual-task (DT) training [8], in which two tasks are performed concurrently [9]. The execution of a DT requires the division of attention and resources between the two tasks [9]. Compared to single-task training, DT exercises train older adults to effectively divide their attention and resources to improve their multitasking ability and integrate their movement capacity and cognitive function [9]. Regular DT training enhances daily function more effectively than single-task training by simulating real-life multitasking scenarios [10]. The most commonly employed forms of DT training include motor–motor, cognitive–cognitive and motor–cognitive tasks [9], which require the simultaneous performance of two motor tasks, two cognitive tasks and a combination of motor and cognitive tasks, respectively [9].

Structured DT training improves both dynamic and static balance and reduces fall in older adults compared to single-task training, multimodal training and usual care [11]. Earlier studies have reported that the DT gait test offers an additional ability to better distinguish between older adults at risk and those not at risk of falls [12, 13]. A recent study highlights seemingly contradicting findings, where both single-task and dual-task gait tests are equally effective in predicting fall risk among community-living older adults [14]. It is important to note that findings pertain to the assessment of gait, rather than evaluating the effects of interventions.

Published studies have reported a low to moderate effect of DT training on fall reduction [11, 15]. However, previous findings regarding improved dynamic balance and functional mobility and reduced falls with DT training are demographically varied, heterogeneous in risk of bias and dependent on training context [16, 17]. Meta-regression analysis addresses these limitations by examining between-study heterogeneity and assessing whether study-level features (e.g., minutes of exercise per week, frequency per week, intervention duration, intensity, challenge, adherence) are linked to superior training effects [16]. To the best of our knowledge, no studies have evaluated between-study heterogeneity and the relationship between higher DT training effects and study-level features, which have been found to improve dynamic balance and functional mobility and prevent falls in older adults [15, 18]. Secondly, the available recent evidence for the efficacy of DT training in reducing falls is limited to participants among community-dwelling older adults [11]. Therefore, there is a need for conducting a subsequent review that explores the benefits of intervention across other study settings, such as old-age homes, hospitals, and other inpatient services. This review thus aimed to (1) compare the effectiveness of DT training with single-task exercise and no exercise in improving dynamic balance and functional mobility and reducing falls among older adults and (2) investigate whether characteristics of specific DT training dosage, including intensity, challenge, adherence and training hours per week, are linked to better improvements in dynamic balance, functional mobility and fall frequency in this population.

## Methods

This systematic review was prospectively registered in PROSPERO (CRD42022334024). This review followed the PRISMA guidelines and is reported in Supplementary file 1.

### Searches

A systematic search of 12 databases (PubMed, Cochrane Library, CINAHL, Embase, MEDLINE, Web of Science, SCOPUS, PEDro, PsycINFO, SafetyLit, CNKI and WanFang Data), a manual search of Clinical Trials (https://clinicaltrials.gov/), International Clinical Trials Registry Platform (https://www.who.int/clinical-trials-registry-platform), the reference lists of recent current controlled trials, a common search engine (Google) and a scholarly search engine (Google Scholar) dated to May 2025 was performed to identify additional studies. No limitations were placed on the publication date. The search strategies employed four themes: “Older adults”, “dual-task”, “balance” or “fall”, and “randomized controlled trial”. The search strategy utilised for PubMed is reported in Supplementary file 2.

### Inclusion and exclusion criteria

Studies were included if (1) they were randomised controlled trials of adults above 60 years of age; (2) the primary intervention was DT training; (3) dynamic balance was assessed using the Berg Balance Scale (BBS) or Fullerton Advanced Balance (FAB) scale, functional mobility was evaluated using the Timed Up and Go (TUG) test, including the dual-task TUG (dTUG), and falls were reported using means, standard deviations, rates or numbers. The search was limited to measures commonly used among older adults. The BBS and FAB scales were frequently used to assess dynamic balance in community-dwelling older adults [19, 20]. The TUG and dual-task TUG were standard measures for evaluating functional mobility in this population [21, 22]. Falls were typically reported as rates,means. numbers, or standard deviations among older adults in community settings [23]; (4) they were published in English or Chinese; (5) they considered the first phase of the intervention outcome in the case of crossover trials; and (6) they compared DT training to single-task exercise, no intervention, usual activity or care, health education classes or social visits.

Studies were excluded if (1) DT training was a component of a blended protocol in which participants received additional interventions (e.g., brain stimulation, dance, music, tai chi); (2) they included older adults with conditions that impair balance, such as stroke, Parkinson’s disease, dementia and other neurodegenerative diseases. Excluding participants with balance impairments ensures a homogeneous study population and isolates the effects of DT training on balance and fall risk in generally healthy older adults. Including participants with such conditions could confound the results, as their balance impairments have different underlying causes and respond differently to interventions compared to healthy older adults [24]; (3) the full text was unavailable; and (4) only a study protocol was available.

### Operational definitions

#### Dual-task (DT)

Performing one task while simultaneously engaging in another task is considered a DT. An example of this is walking while talking on the phone. This review examined three DT types: motor–cognitive, motor–motor and cognitive–cognitive.

#### Falls

“A fall is defined as an event which results in a person coming to rest inadvertently on the ground or floor or other lower level (WHO)” [25].

#### Intensity

Intensity is defined as the energy required to complete a DT within a specified period. Intensity was categorised using metabolic equivalents of task (METs). One MET corresponds to the activity per minute required when sitting at rest (1 MET = 3.5 mL/kg/min). Intensity was categorized 1.6–2.9 METs have mild intensity, 3.0–5.9 METs have moderate intensity and ≥ 6.0 METs have high intensity [26]. Mild-intensity exercise includes light walking, moderate-intensity exercise includes ascending stairs and using a wheeled walker, and high-intensity exercise includes running or carrying heavy loads upstairs [26]. Additional information regarding the exercises used to estimate METs can be found in Table 1.

**Table 1:** General description of the included study

Authors	Country/continent	Study settings/type of dual task/male/female/total participants/ age(mean)/MET	Exercises/sets/duration per session(mins)/sessions per week/total duration (TD) per week (mins)/period of intervention(weeks)		Assessment (weeks) (post/ follow-up)	PEDro score	Outcome
			Intervention	Control			
Aragao-Santos et al. (2024) [55]	Brazil/ South America	Study settings: DT type: Motor-cognitive M: 0 F: 61 Total: 61 Age: (67) MET: 4.3	Mt: Mobility and stability training, static and dynamic balance training, ball passing/ Cg: Working memory, naming/ Sets: - Dur/s(min): 50 S/week: 3 TD/week: 150 Int (weeks): 16 tmDT:	Mt: Joint mobility and stability training, muscle power training, strength exercise (pull, push, squat, transportation) Cg: Sets: Dur/s(min): 50 S/week: 3 TD/week: 150 Int (weeks): 16 tmDT:	Post: 16 Follow-up: 12	8	TUG
Balci et al.(2022) [54]	Turkey/ Asia	Study settings: General community dwellers DT type: Motor-cognitive M: 6 F: 39 Total: 45 Age: (73) MET: 6	Mt: Static and dynamic balance exercise/ Cg: visual attention tasks, auditory attention tasks, planning tasks, verbal fluency, simple mental math, maze activities/ Sets: - Dur/s(min): 30 S/week: 3 TD/week: 90 Int (weeks): 4 tmDT:	Mt: Static and dynamic balance exercise Cg: Sets: Dur/s(min): 30 S/week: 3 TD/week: 90 Int (weeks): 4 tmDT:	Post: 4	6	BBS
Brustio et al. (2017) [49]	Italy / Europe	Study settings: General community dwellers DT type: Motor-motor/ M: 18 F: 42 Total: 60 Age: (74.4) MET: 3.0	Mt: Walk and turn, or walk backwards and forwards while wearing a sweater, buttoning and unbuttoning a shirt Sets: - Dur/s(min): 60 S/week: 2 TD/week: 120 Int (weeks): 6 tmDT:	Group1: Mt: Semi-tandem stand, tandem stand, or one leg stance, walking exercise Sets: - Dur/s(min): 60 S/week: 2 TD/week: 120 Int (weeks): 16 tmDT: Group 2: No intervention	Post: 16	7	TUG
Castillo de Lima et al. (2023) [44]	Brazil/ South America	Study settings: General community dwellers DT type: Motor-cognitive/ M: 5 F: 11 Total: 16 Age: (65.9) MET: 5.5	Mt: Agility training/ Cg: words speaking/ Sets: 1—4 Dur/s(min): 30 S/week: 2 TD/week: 60 Int (weeks): 14 tmDT:	Mt: Agility training Cg: Sets: 1—4 Dur/s(min): 30 S/week: 2 TD/week: 60 Int (weeks): 14 tmDT:	Post: 14	6	TUG
da Silva et al. (2021) [45]	Brazil / South America	Study settings: General community dwellers/ DT type: Motor-cognitive/ M: 2 F: 14 Total: 16 Age: (70.8) MET: 4.3	Mt: Reach for a ball that the instructor alternates in different directions, single-leg stance Cg: Naming colors/days of the week/names, when a green card is shown, individuals raise their hands Sets: Dur/s(min): 60 S/week: 3 TD/week: 180 Int (weeks): 6 tmDT:	Mt: Reach for a ball that the instructor alternates in different directions, single-leg stance Sets: Dur/s(min): 60 S/wk: 3 TD/week: 180 Int (weeks): 6 tmDT:	Post: 6	6	TUG
de Oliveira et al.(2021) [41]	Brazil/ South America	Study settings: General community dwellers DT type: Motor-cognitive M: 5 F: 43 Total: 49 Age: (68) MET: 6	Mt: unstable strength training (leg press 45^。^, horizontal dumbbell chest press, unilateral row with dumbbell, plank, abdominal, bridge) Cg: random number generation, word association, backward recitation, working memory Sets: (month 1: 2, month 2–3: 3, month 4–5: 4, month 6: 5) Dur/s(min): 60 S/week: 3 TD/week: 180 Int (weeks): 24 tmDT:	Mt: unstable strength training (leg press 45^。^, horizontal dumbbell chest press, unilateral row with dumbbell, plank, abdominal, bridge) Sets: (month 1: 2, month 2–3: 3, month 4–5: 4, month 6: 5) Dur/s(min): 60 S/week: 3 TD/week: 180 Int (weeks): 24 tmDT:	Post: 24	7	BBS, TUG
Desjardins-Crépeau et al. (2016) [46]	Canada / North America	Study settings: General community dwellers DT type: Motor-cognitive M:53 F:13 Total: 66 Age: (72.4) MET: 4.6	Group1: Mt: Treadmill training/ Cg: Number discrimination, shape discrimination Sets: Dur/s(min): 60 S/week: 3 TD/week: 180 Int (weeks): 12 Group2: Mt: Back scratch, back arm press, standing thigh, overhead back Cg: Number discrimination, shape discrimination Sets: 10 Dur/s(min): 60 S/wk: 3 TD/week: 180 Int (weeks): 12 tmDT:	Cg: Introductory exercises to computers and diverse software/ Sets: Dur/s(min): 60 S/wk: 3 TD/week: 60 Int (weeks): 12 tmDT:	Post: 12	5	TUG
Duque et al. (2013) [81]	Australia / Oceania	Study settings: General community dwellers DT type: Motor-cognitive M:27 F:43 Total: 70 Age: (79.3) MET: 4	Mt-Cg: Using VR for visual search tasks, such as letters Sets: Dur/s(min): 30 S/wk: 2 TD/week: 60 Int (weeks): 6 tmDT: VR system (Balance ­ Rehabilitation Unit)	No intervention	Post: 6 Follow-up:39	5	Fall
Franco et al.(2012) [56]	USA/ North America	Study settings: Residential care dwellers DT type: Motor-cognitive M: 7/ F: 25/ Total: 32/ Age: (80) MET: 5	Mt: Wii fit balance training (heading soccer, ski jumping, ski slalom, tightrope, table tilt, balance bubble) and supplemental home exercise Cg: Wii fit exercise Sets: Dur/s(min): 13 S/week: 2 TD/week: 26 Int (weeks): 3 tmDT: Nintendo Wii Fit	Mt: matter of balance exercise/ no intervention Cg: Sets: Dur/s(min): 13 S/week: 2 TD/week: 26 Int (weeks): 3 tmDT:	Post: 3	6	BBS
Granacher et al. (2021) [72]	Germany/ Europe	Study settings: General community dwellers DT type: Motor-cognitive M: 24 F: 27 Total: 51 Age: (65.7) MET: 3.8	Mt: tooth brushing + balance exercise Cg: - Sets: Dur/s(min): 6 S/week: 7 TD/week: 42 Int (weeks): 8 tmDT:	No intervention	Post: 8	6	TUG
Gschwind et al. (2014) [73]	Australia/ Oceania	Study settings: General community dwellers DT type: Motor-cognitive M: 60 F: 93 Age: (74.7) MET: 4.0	Mt: Walking, weight shifting, knee bending, stepping in different directions/ Cg: Remembering objects/ Sets: Dur/s(min): 40 S/week: 3 TD/week: 120 Int (weeks): 16 tmDT: Exergame	Educational booklet	Post: 16	8	TUG
Hinman.(2002) [57]	USA/ North America	Study settings: General community dwellers DT type: Motor-cognitive/ M: 33 F: 55 Total: 88 Age: (72) MET: 5	Mt: Biodex balance system training Cg: Computerized training Sets: Dur/s(min): 20 S/week: 3 TD/week: 60 Int (weeks): 4 tmDT: Biodex Balance System	No intervention	Post: 4	5	BBS
Hiyamizu et al. (2012) [47]	Japan/ Asia	Study settings: General community dwellers DT type: Motor-cognitive M: 17 F: 26 Total: 43 Age: (72) MET: 1	Mt: strength and balance training/ Cg: calculation, visual, verbal tasks/ Sets: Dur/s(min): 60 S/week: 2 TD/week: 120 Int (weeks): 12 tmDT:	Mt: Strength & balance training Sets: Dur/s(min): 60 S/week: 2 TD/week: 120 Int (weeks): 12 tmDT:	Post: 12	7	TUG
Htut et al.(20082018) [42]	Thailand/ Aisa	Study settings: Residential care dwellers DT type: Motor-cognitive M: 47 F: 37 Total: 63 Age: (76) MET: 5	Mt: VR exercise (X-box 360) 6 games involving upper and lower limb movements and balance training Cg: VR game training Sets: Dur/s(min): 30 S/week: 3 TD/week: 90 Int (weeks): 8 tmDT: VR	Mt: Strength and balance exercises), Group: no intervention Sets: - Dur/s(min): 30 S/week: 3 TD/week: 90 Int (weeks): 8 tmDT:	Post: 8	7	BBS
Javadpour et al. (2022) [78]	Iran/ Asia	Study settings: Residential care dwellers DT type: Motor-cognitive M: 20 F: 49 Total: 69 Age: (68.6) MET: 3.8	Mt: balance training (standing, walking) Cg: naming, counting back Sets: Dur/s(min): 50 S/week: 3 TD/week: 150 Int (weeks): 6 tmDT:	Mt: Balance training (standing and walking) Sets: Dur/s(min): 50 S/week: 3 TD/week: 150 Int (weeks): 6 tmDT:	Post: 6	8	FAB
Lai et al. (2013) [58]	Taiwan/ Asia	Study settings: General community dwellers DT type: Motor-cognitive/ M: 13 F: 15 Total: 30 Age: (71) MET: 5	Mt: Interactive video-game-based training Cg: Video game training Sets: Dur/s(min): 30 S/week: 3 TD/week: 90 Int (weeks): 6 tmDT: Video game	No intervention	Post: 6 Follow-up: 12	4	BBS, TUG
Lee. (2020) [59]	South Korea/ Asia	Study settings: General community dwellers DT type: Motor-cognitive M: 31/ F: 25/ Total: 56 Age: (80) MET: 5	Mt: VR gait training, non-motorized treadmill training/ Cg: VR training/ Sets: Dur/s(min): 50 S/week: 5 TD/week: 250 Int (weeks): 4 tmDT: VR	Mt: treadmill training/ Cg: -/ Sets: -/ Dur/s(min): 50 S/week: 5 TD/week: 250 Int (weeks): 4 tmDT:	Post: 4	7	BBS
Mirelman et al. (2016) [82]	Israel / Asia	Study settings: General community dwellers DT type: Motor-cognitive M: 182 F: 100 Total: 282 Age: (73.75) MET: 6	Mt-Cg: Using VR system to provide virtual environment for treadmill training Sets: Dur/s(min): 45 S/week: 3 TD/week: 135 Int (weeks): 6 tmDT: VR treadmill training	Mt: Treadmill training Sets: Dur/s(min): 45 S/week: 3 TD/week: 135 Int (weeks): 6 tmDT:	Post: 6	6	Fall
Morat et al. (2019) [60]	Germany/ Europe	Study settings: General community dwellers DT type: Motor-cognitive M: 28 F: 17 Total: 45 Age: (69) MET: 4	Mt: Volitional stepping exergames with unstable conditions Cg: attention, working memory, mental rotation, cognitive flexibility Sets: Dur/s(min): 40 S/week: 3 TD/week: 120 Int (weeks): 8 tmDT: exergame	No intervention	Post: 8	5	TUG
Nascimento et al. (2023) [61]	Brazil/ South America	Study settings: General community dwellers DT type: Motor-cognitive M: 0 F: 44 Total: 44, Age: (66.2) MET: 6	Mt: gait, static, and dynamic balance training Cg: counting, memorization, ordering, stroop tasks Sets: Dur/s(min): 60 S/week: 2 TD/week: 120 Int (weeks): 12 tmDT:	No intervention	Post: 12 Follow-up: 12	9	TUG
Nematollahi et al. (2016) [79]	Iran/ Asia	Study settings: Residential care dwellers DT type: Motor-cognitive M: 12 F: 32 Total: 44 Age: (65.4) MET: 4	Mt: Postural task (narrow-base walking, kicking a ball) Cg: Counting, naming Sets: Dur/s(min): 60 S/week: 3 TD/week: 180 Int (weeks): 4 tmDT:	Mt: Conventional training group – sitting on balance ball, standing, and moving task; Multisensory training group – sitting on balance ball with closed eyes or walking on foam surface, single leg standing with diagonal head movements Sets: Dur/s(min): 60 S/week: 3 TD/week: 180 Int (weeks): 4 tmDT:	Post: 4	5	FAB
Norouzi et al. (2019) [62]	Iran/ Asia	Study settings: DT type: Motor-cognitive Motor-motor M: 40 F: 0 Total: 40 Age: (68) MET: 5.8	Mt: resistance training (isokinetic exercise device) + simultaneous motor training (throwing ball up and down, a bag, holding a bag, balancing a cup)/ Cg: matching, counting, mental arithmetic, spelling, remembering/ Sets: Dur/s(min): 80 S/week: 3 TD/week: 240 Int (weeks): 4 tmDT:	No intervention	Post: 4 Follow-up: 12	6	BBS
Park et al. (2015) [64]	South Korea/ Asia	Study settings: General community dwellers DT type: Motor-cognitive M: 19 F: 5 Total: 24 Age: (66.5) MET: 7	Mt: VR exercise training (Wii-Fit balance program: Soccer heading, snowboard slalom, table tilt) Cg: VR game training Sets: Dur/s(min): 30 S/week: 3 TD/week: 90 Int (weeks): 8 tmDT: VR game	Mt: ball exercise (bouncing, pelvic tilting, pelvic circle) Sets: Dur/s(min): 30 S/week: 3 TD/week: 90 Int (weeks): 8 tmDT:	Post: 8	4	TUG
Park (2022) [50]	South Korea/ Asia	Study settings: General community dwellers DT type: Motor-cognitive M: F: Total: 58 Age: (71.4) MET: 3.8	Mt: walking, stepping over obstacle Cg: spelling, naming, counting Sets: 1 Dur/s(min): 45 S/week: 2 TD/week: 90 Int (weeks): 6 tmDT:	Mt: stability training (standing, walking, throwing, and catching a ball) Sets: 1 Dur/s(min): 45 S/week: 2 TD/week: 90 Int (weeks): 6 tmDT:	Post: 6	7	TUG
Padala et al. (2017) [63]	USA/ North America	Study settings: General community dwellers DT type: Motor-cognitive M: 26 F: 4 Total: 30 Age: (68) MET: 5.3	Mt: Wii-fit exercise (balance, yoga, strength training, aerobic, cycling) Cg: Wii Fit exericise Sets: - Dur/s(min): 45 S/week: 3 TD/week: 135 Int (weeks): 8 tmDT: Wii Fit	Mt: - Cg: brain fitness computer program Sets: Dur/s(min): 45 S/week: 3 TD/week: 135 Int (weeks): 8 tmDT: computer-based cognitive exercise	Post: 8	7	BBS
Phirom et al. (2020) [48]	Thailand / Asia	Study settings: General community dwellers DT type: Motor-cognitive M: 7 F: 33, Total: 40 Age: (69.8) MET: 3.5	Mt: Step on the target presented, collect dropping objects into the basket / Cg: Recall questions regarding the story’s/ Sets: Dur/s(min): 60 S/week: 3 TD/week: 180 Int (weeks): 12 tmDT: Exergame	Educational material covering cognitive enhancement and fall prevention strategies	Post: 12	6	TUG
Plummer-D'Amato et al. (2012) [74]	USA / North America	Study settings: General community dwellers DT type: Motor-cognitive M: 1 F: 18 Total: 19 Age: (74.6) MET: 4.0	Mt: Standing and walking on a foam balance beam, lateral stepping, toe tapping on step/ Cg: Randomly naming numbers between 100 and 500, spelling backwards 3-letter words Sets: Dur/s(min): 45 S/week: 1 TD/week: 45 Int (weeks): 4 tmDT:	Mt: Standing and walking on a foam balance beam, lateral stepping, toe tapping on step/ Sets: Dur/s(min): 45 S/week: 1 TD/week: 45 Int (weeks): 4 tmDT:	Post: 4	7	TUG
Pradhan et al. (2018) [66]	India / Asia	General community dwellers DT type: Motor-cognitive M: 18 F: 22 Total: 40 Age: (69.8) MET: 4.0	Mt: Walk within 2 parallel strips marked on the floor of 4 m distance, walk and step over 3 obstacles/ Cg: Counting backward by threes from any starting number from 90 to 200/ Sets: Dur/s(min): 45 S/week: 3 TD/week: 135 Int (weeks): 4 tmDT:	Mt: Walk within 2 parallel strips marked on the floor of 4 m distance, walk and step over 3 obstacles Sets: Dur/s(min): 45 S/wk: 3 TD/wk: 135 Int (wks): 4 tmDT:	Post: 4	6	BBS
Reve & de Bruin. (2014) [83]	Switzerland / Europe	Study settings: Residential care dwellers DT type: Motor-cognitive M: 55 F: 101 Total: 156 Age: (81.5) MET: 4	Mt: One-legged stance training, tandem standing and walking, walking on heels, backward and sideward walking, turns / Cg: Playing computer games, such as a motorcycle is driven along a road, and the participant’s task was to react as quickly as possible when obstacles appear Sets: Dur/s(min): 40 S/week: 2 TD/week: 80 Int (weeks): 12 tmDT: Computer game	Mt: One-legged stance training, tandem standing and walking, walking on heels, backward and sideward walking, turns Sets: Dur/s(min): 40 S/week: 2 TD/week: 80 Int (weeks): 12 tmDT: Computer game	Post: 12	5	Fall
Rezola-Pardo et al. (2022) [76]	Spain / Europe	Study settings: Nursing home residents DT type: Motor-cognitive M: 28 F: 57 Total: 85 Age: (84.8) MET: 2.3	Mt: Chair stand, one-legged stand, standing on tips and heels, ball reaching Cg: Naming animals, professions, or even dog breeds Sets: Dur/s(min): 60 S/wk: 2 TD/wk: 120 Int (wks): 13 tmDT:	Mt: Chair stand, one-legged stand, standing on tips and heels, ball reaching Sets: Dur/s(min): 60 S/week: 2 TD/week: 120 Int (weeks): 13 tmDT:	Post: 13	7	TUG
Rosado et al. (2021) [80]	Portugal / Europe	Study settings: General community dwellers DT type: Motor-cognitive M: 6 F: 42 Total: 48 Age: (75.3) MET: 2.8	Group1: Mt: Moving around cones with a fit ball as fast as possible, forward and backward / Cg: drawing a 3, 8 and a Z on the floor, reciting the days of the week backwards while walking backwards Sets: Dur/s(min): 75 S/week: 3 TD/week: 225 Int (weeks): 24 tmDT: Group2: Mt: Moving around cones with a fit ball as fast as possible, forward and backward / Cg: drawing a 3, 8, and a Z on the floor, reciting the days of the week backwards while walking backwards Sets: Dur/s(min): 75 S/week: 3 TD/week: 225 Int (weeks): 24 tmDT:	No intervention	Post: 24 Follow-up: 12	6	TUG, Fall
Rosado et al. (2022) [51]	Portugal / Europe	Study settings: General community dwellers DT type: motor-cognitive M: 6 F: 42 Total: 48 Age: (75) MET: 2.8	Group1: Mt: Standing up and sitting down from the chair, fit ball wall squats Cg: Countdown by 3 from 30, reciting their phone number backwards Sets: Dur/s(min): 75 S/week: 3 TD/week: 225 Int (weeks): 24 tmDT: Group2: Mt: Standing up and sitting down from the chair, fitball wall squats, stand up on the vibration platform without shoes while holding the handlebar with bent knees/ Cg: Countdown by 3 from 30, reciting their phone number backwards Sets: Dur/s(min): 75 S/week: 3 TD/week: 225 Int (weeks): 24 tmDT:	No intervention	Post: 24 Follow-up: 12	7	Fall
Rose & Clark. (2000) [67]	USA /North America	Study settings: General community dwellers DT type: Motor-motor M: 13 F: 28 Total: 41 Age: (78.7) MET: 4.0	Mt-Mt: Hand-to-hand catching while stepping, stepping to targets while catching thrown object Sets: Dur/s(min): 45 S/week: 2 TD/week: 90 Int (weeks): 8 tmDT: Computerized Pro Balance Master	No intervention	Post: 8	3	BBS
Park et al. (2014) [65]	South Korea/ Asia	Study settings: Residential care dwellers DT type: Motor-cognitive M: 0 F: 30 Total: 30 Age: (75) MET: 7	Mt: Ski Slalom Wii sports and Soccer heading program/ Cg: Wii training Sets: Dur/s(min): 30 S/week: 3 TD/week: 90 Int (weeks): 6 tmDT: VR	Mt: balance training (hip, knee and ankle joint training, lower limbs training) Sets: 3 Dur/s(min): 30 S/week: 3 TD/week: 90 Int (weeks): 6 tmDT:	Post: 6	5	BBS
Silsupadol et al. (2009) [68]	USA / North America	Study settings: General community dwellers residents DT type: Motor-cognitive M: 1 F:18 Total: 19 Age: (74.8) MET: 4.0	Mt: Standing on foam with rapid alternating hand movement, throwing and catching a ball, and tandem standing while holding a basket/ Cg: Naming objects, remembering numbers Sets: Dur/s(min): 45 S/week: 3 TD/week: 135 Int (weeks): 4 tmDT:	Mt: Standing with eyes closed, tandem standing, and standing on compliant surfaces/ Sets: Dur/s(min): 45 S/week: 3 TD/week: 135 Int (weeks): 4 tmDT:	Post: 4	8	BBS
Smith-Ray et al. (2014) [43]	USA / North America	Study settings: General community dwellers DT type: Cognitive-cognitive M:45/ F:41/Total: 86/ Age: (72.5) MET: 4.0	Cg-Cg: Select vehicle type; then identifies road sign's original location among surrounding cars Sets: Dur/s(min): 60 S/week: 2 TD/week: 120 Int (weeks): 10 tmDT: computer-based exercise	No intervention	Post: 10	6	BBS
Smith-Ray et al. (2015) [52]	USA / North America	Study settings: General community dwellers DT type: Cognitive-cognitive M: 12 F: 39 Age: (81.9) MET: 4.0	Mt-Cg: Select vehicle type; then identifies road sign's original location among surrounding cars Sets: Dur/s(min): 60 S/week: 3 TD/week: 180 Int (weeks): 10 tmDT: Computer game	Given two brochures on fall prevention for older adults/ tmDT: computer game	Post: 10	6	TUG
Sturnieks et al. (2024) [31]	Austria / Australasia	Study settings: General community dwellers DT type: Motor-cognitive M: 220 F: 549 Total: 769 Age: (72.5) MET: 4.3	Mt-Cg: Play smart ± step system games while standing and taking quick and appropriate steps on the mat Sets: Dur/s(min): S/week: TD/week: 88 Int (weeks): 26 tmDT: exergame	Control 1: Cg: Seated and using the hands to press sensor targets on the desktop touch pad Sets: Dur/s(min): S/week: TD/week: 88 Int (weeks): 26 tmDT: Control 2: Phone call visit	Post: 26	6	TUG, Fall
Szturm et al. (2011) [69]	Canada / North America	Study settings: General community dwellers DT type: Motor-cognitive M:11 F:19 Total: 30 Age: (80.7) MET: 4.0	Mt-Cg: Standing on a pressure mat in front of the screen, shift weight to move a game sprite (flower) left to right on the display in order to catch an object (bee) falling from the top of the screen Sets: - Dur/s(min): 45 S/wk: 2 TD/week: 90 Int (weeks): 8 tmDT: computer game	Mt: Side-leg raises, squats, and standing up from a chair and sitting down in the chair without using hands Sets: Dur/s(min): 45 S/week: 2 TD/week: 90 Int (weeks): 8 tmDT:	Post: 8	5	BBS
Talwar et al. (2015) [75]	India / Asia	Study settings: Residential care dwellers DT type: Motor-motor M:11 F:19 Total: 30 Age: (80.7) MET: 6.0	Mt-Mt: Walking movements in multiple directions while passing the ball / Sets: Dur/s(min): 60 S/week: 3 TD/week: 180 Int (weeks): 4 tmDT:	Mt: Walking movements in multiple directions, forward, backward, lateral, and oblique steps Sets: Dur/s(min): 60 S/week: 3 TD/week: 180 Int (weeks): 4 tmDT:	Post: 4	3	BBS
Yamada et al. (2011) [53]	Japan / Aisa	Study settings: General community dwellers DT type: Motor-cognitive M: 12 F: 41 Total: 53 Age: (80.8) MET: 6.0	Mt: Stepped up and down alternating between left and right legs as quickly as possible while returning the legs to the initial starting position/ Cg: Enumerating words within a category or letter Sets: Dur/s(min): 50 S/week: 1 TD/week: 50 Int (weeks): 24 tmDT:	Mt: Stepped up and down alternating between left and right legs as quickly as possible while returning the legs to the initial starting position Sets: Dur/s(min): 50 S/week: 1 TD/week: 50 Int (weeks): 24 tmDT:	Post: 24	7	TUG
Yeşilyaprak et al. (2016) [70]	Turkey / Europe	Study settings: Nursing home residents DT type: Motor-cognitive M: 6 F: 12 Total: 18 Age: (70.1) MET: 6.0	Mt-Cg: Follow the onscreen visual displays and listen to audio feedback (pointing the right figure) while maintaining their stability during balance activities in a standing posture Sets: Dur/s(min): 45 S/week: 3 TD/week: 135 Int (weeks): 6 tmDT: VR	Mt: Walking forward 10 steps and pivoting 180^0^, alternate single leg stands, walking forward, sideways, and backwards/ Sets: Dur/s(min): 45 S/week: 3 TD/week: 135 Int (weeks): 6 tmDT:	Post: 6	5	BBS
Zahedian-Nasab et al. (2021) [71]	Iran / Asia	Study settings: Nursing home residents DT type: Motor-cognitive M: 44 F:16 Total: 60 Age: (69.7) MET: 6.0	Mt-Cg: Weight shifting to the right and left or up and down, by using the Xbox Kinect sports pack Sets: Dur/s(min): 40 S/week: 2 TD/week: 80 Int (weeks): 6 tmDT: VR (Xbox)	Mt: Jogging in the nursing home, table tennis, and some artistic activities Sets: Dur/s(min): S/week: TD/week: Int (weeks): 6 tmDT:	Post: 6	6	BBS
Zheng et al. (2013) [77]	China / Asia	Study settings: Residential care dwellers DT type: Motor-cognitive M: 47 F: 53 Total: 100 Age: (68.1) MET: 4.0	Mt: One leg stance, two leg stance, jogging end to end, sideways walking, running in a zigzag line, and backward walking/ Cg: Response to continuous simple addition/subtraction questions (such as 3 + 2 = 5, 100–7 = 93) Sets: Dur/s(min): 40 S/week: 3 TD/week: 120 Int (weeks): 8 tmDT:	Mt: Marching on the spot, knee lifts, heel digs, shoulder rolls, knee bends Sets: Dur/s(min): 40 S/week: 3 TD/week: 120 Int (weeks): 8 tmDT:	Post: 8	6	FAB

*MET* Metabolic equivalent, *1 MET* 3.5 mL/kg/min, *Dur* Duration, *Mt* Motor, *Cg* cognitive, *Int* Intervention, *tmDT* Technology-mediated dual-task, *BBS* Berg Balance Scale, *FAB* Fullerton advanced balance scale, *TUG* Time up and go, *F* Female, *M* Male, *DT* Dual-task, *S* Session, *TD* Total duration, *Int* Period of intervention

#### Challenge

Challenge refers to the level of difficulty of the DT training. The challenge level was classified based on the difficulty of the secondary motor or cognitive task. For motor tasks, difficulty was increased by (1) altering the centre of gravity, (2) reducing upper limb support, (3) decreasing visual feedback, (4) reducing the base of support, and (5) disrupting the supporting surface. Motor tasks were categorised as mildly challenging if one of these conditions was adjusted, moderately challenging if two conditions were adjusted, and highly challenging if three or more conditions were adjusted. In no particular order of difficulty, the cognitive tasks, included the following: (1) verbal fluency, (2) visual search exercises, (3) arithmetic exercises, (4) recall and memory exercises, and (5) information processing speed. Simialr to the motor tasks, mild challenge were those adjusting any one of these cognitive conditions, moderately challenging if two conditions were adjusted, and highly challenging if three or more conditions were adjusted.

#### Adherence rate

Adherence rate is the percentage of sessions attended compared to the total number of sessions offered. In this review, studies were considered to have excellent adherence if participants attended 95% or more of sessions, good adherence if participants attended 90%–94% of sessions and poor adherence if participants attended < 90% of sessions.

#### Data extraction

First, all titles were evaluated by a single reviewer. Two reviewers were then responsible for screening the abstracts and full texts. Four steps were employed to screen the studies. Disagreements were settled through discussion. If the two reviewers were unable to reach a consensus, a third reviewer was consulted. Two authors independently conducted data extraction. Data from the included studies were extracted and summarised in Excel before being imported into STATA for analysis.

### Quality assessment

The included studies’ methodological quality was assessed using the Physiotherapy Evidence Database (PEDro) scale [27]. The derived PEDro scores were used in the meta-regression [16]. Available PEDro scores were retrieved from the PEDro database and one reviewer manually determined scores for any studies not included on the PEDro website. As meta-regression analysis requires a numerical score for methodological quality, the PEDro score was preferred over other methodological quality rating tools [16]. In subgroup analysis, the PEDro score was used to measure the random effect size of high-quality studies. A study’s methodological quality was categorised as high if the PEDro score was ≥ 6, moderate if the PEDro score was 4–5 and low if the PEDro score was ≤ 3 [28].

Risk of bias in the included studies was evaluated using the Cochrane risk of bias tool [29], which rates studies as having low, some concern or high risk of bias [29]. Studies were classified as having low risk of bias if all domains had low risk, some concern for risk of bias if one or more domains had some concern and high risk of bias if one or more domains had high risk [29].

The transparent Grading of Recommendations, Assessment, Development, and Evaluations (GRADE) approach was utilised to report the quality of the evidence [29]. The strength of the evidence was rated as either "strong," "moderate," "low," or "very low" according to GRADE [29]. The strength of the evidence was rated as either high, moderate, low or very low according to the GRADE tool, which assesses evidence quality based on five domains: indirectness, inconsistency, publication bias, imprecision and risk of bias [29]. The GRADE rating was decreased by one or two levels in studies with “serious concern” or “very serious concern” in any of the five domains [29]. The GRADE rating was upgraded if trials were well-designed and reported significant effect sizes, dose–response relationships or convincing evidence [29].

### Outcome measures

Falls is measured using both retrospective recall and prospective recording methods [30]. Falls are monitored through participant self-reports collected during monthly follow-up interviews [31]. The self-reported data is collected using monthly diaries and a questionnaire [32]. This method is often used in fall prevention studies for its feasibility and low cost [33].

The BBS is a 14-item objective tool that assesses dynamic balance [34]. Each item is scored from 0 to 4, with total scores ranging from 0 to 56 and higher scores indicating better balance [34]. Completing the BBS requires approximately 20 min. The BBS is suggested as a standard measure of dynamic balance in older adults, with excellent inter-rater reliability (ICC = 0.99; CI: 0.988–0.996) and concurrent validity (*r* = 0.84; *P* < 0.001) [34].

The FAB scale is a 10-item performance-based tool that evaluates dynamic balance [20]. Each item is scored from 0 to 4, with total scores ranging from 0 to 40. Participants complete 10 actions that test diverse components of dynamic balance, including standing with eyes closed, reaching forward, turning, walking and balancing on one leg. Higher scores indicate better balance [20]. The FAB scale requires approximately 12 min to complete and is suggested as a standard measure of dynamic balance in older adults due to its excellent test–retest reliability (ICC = 0.96; *P* < 0.001) and concurrent validity (*r* = − 0.75; *P* < 0.05) [20].

The TUG assesses functional mobility and is recorded in seconds [35]. The TUG is a timed task requiring the participant to stand up from a chair, walk 3 m, turn 180 degrees and return to the chair [35]. Shorter times to complete the task indicate better functional mobility. The TUG is suggested as a standard measure of functional mobility in older adults, with high inter-rater reliability (ICC = 0.97; CI: 0.66–0.82) and acceptable concurrent validity (*r* = − 0.88; *P* < 0.001) [35, 36]. The dTUG assesses DT ability and is similarly recorded in seconds [37]. The testing procedure is identical to the conventional TUG except that the participant performs a cognitive task such as a mental calculation while completing the TUG [37]. Shorter completion times indicate better DT ability [37]. The dTUG is suggested as a standard measure of DT ability in older adults, with excellent test–retest reliability (rT1–T2 = 0.98) and concurrent validity (*r* = − 0.66; *P* < 0.001) [37].

### Statistical analyses

Relevant data, including study design, sample demographics, DT training design and intervention effects, were extracted from each study. Study design information included sample size, follow-up period and PEDro score; sample demographics included mean age, DT training design included training type, training hours per week, training intensity, challenge level and intervention adherence; intervention effects included number of falls and means and standard deviations for the BBS, FAB scale, TUG and dTUG. For studies that did not provide raw mean and standard deviation, standard error of the mean, or median and interquartile range data, numerical data were obtained from the study’s graphs or figures using an online computer program (https://apps.automeris.io/wpd/).

The statistical cutoff value for the level of significance was *P* ≤ 0.05, the confidence interval (CI) was 95%, heterogeneity greater than 50% was considered high in the meta-analysis and meta-regression and a regression coefficient between 0 and 1 was considered significant [16]. STATA version 17 (Stata Corp LLC, College Station, TX, USA) was used for the random-effects meta-analysis and meta-regression. The random-effects model was employed due to its better results for high heterogeneity and small sample sizes (< 30) [38]. Covariates were not included in the meta-regression analysis.

Subgroup analysis was conducted to test the stability of the results of the random-effects meta-analysis. To minimise the small-study effect of DT training on dynamic balance, motor function and falls, a random effect size in the subgroup analysis was utilised [39]. The subgroup analysis included the following components: 1) sample size, distinguishing between smaller samples (fewer than 30 participants at randomisation) and larger samples (30 or more participants at randomisation), as fewer than 30 participants may limit statistical power and increase the risk of type II errors [40]; 2) methodological quality, categorised as lower quality (PEDro score < 6) and higher quality (PEDro score ≥ 6) 3) instructor- or physiotherapist-guided interventions; 4) comparator type, including active, control and usual care; 5) use of technology-mediated DT (tmDT) training; 6) use of conventional DT exercises without technological components and 7) DT type, encompassing motor–motor, cognitive–cognitive and motor–cognitive exercises. Subgroup analysis was conducted to explore how the impact of DT training on dynamic balance, functional mobility and falls varied among subgroups.

Only the BBS and TUG were considered for meta-regression, as the other scales did not meet the criterion of having a minimum of 10 studies [29]. The regression coefficient obtained from meta-regression analysis describes how the intervention effect changes with a one-unit increase in the potential effect modifiers. A statistically significant regression coefficient explains the linear relationship between the intervention effect and the explanatory variables [29]. Meta-regression analysis was performed to evaluate the effects of study-level features corresponding to study design (sample size, follow-up period, PEDro score, instructor guidance, physiotherapist guidance and exercise tailored to participnats), sample characteristics (mean age), training features (minutes of exercise per week; frequency per week; intervention duration; mild, moderate or high intensity of DT training; mild, moderate or high challenge level of DT training; and excellent adherence to DT training (95% of treatment sessions were attended by 100% of participants) [16].

A visual examination of funnel plots and Egger’s test were used to evaluate the effects of small studies. The mean difference (MD) determined in the meta-analysis was compared with the minimal clinically important difference (MCID) for the given outcome measure to explore if any changes were clinically meaningful.

## Results

**Fig. 1:**
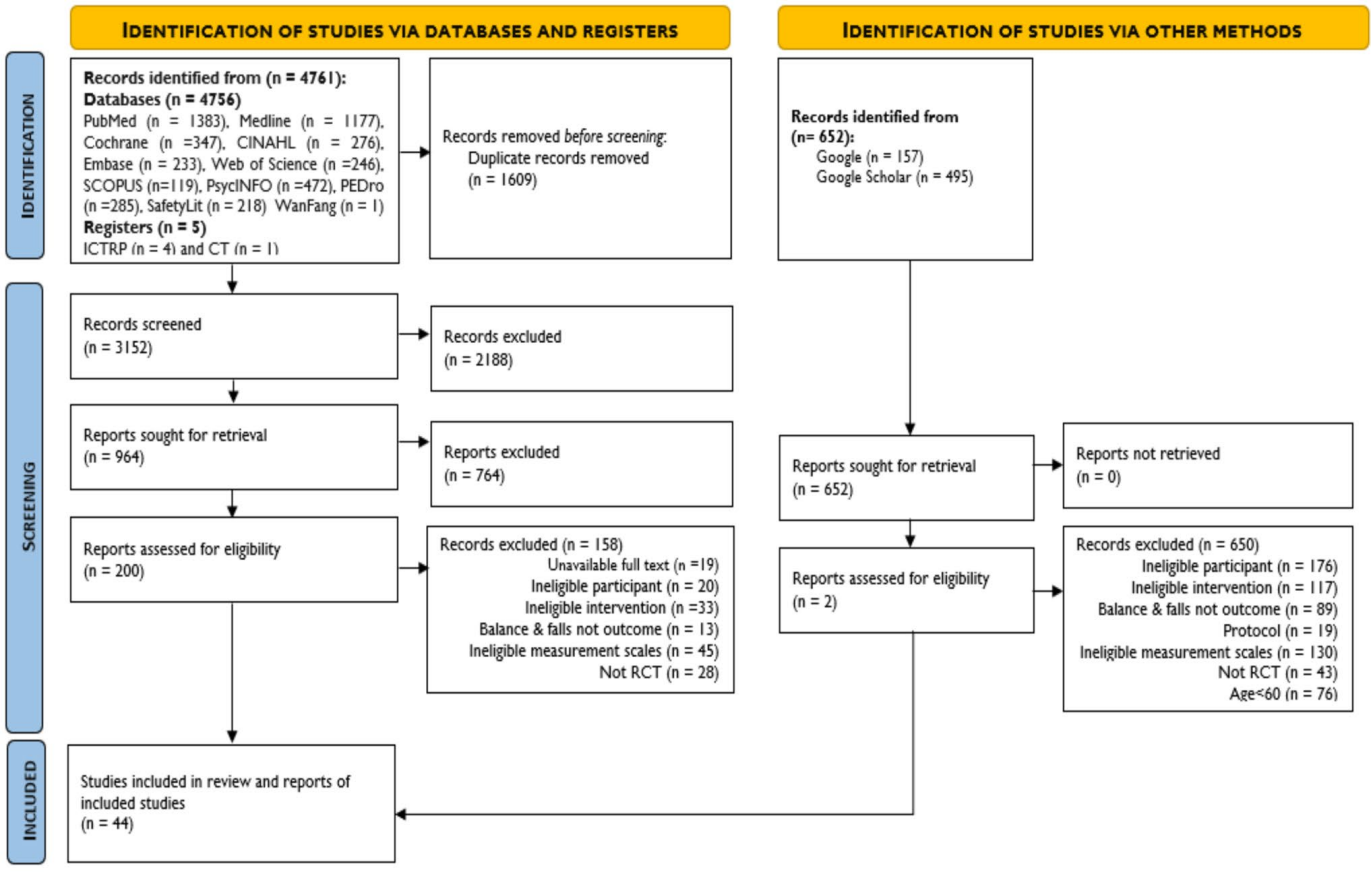
Flow diagram for systematic search process of databases, registers and other sources

The database search yielded 2418 studies. After screening, 44 studies (dynamic balance (*n* = 21), functional mobility (*n* = 21) and falls (*n* = 6)) met the inclusion criteria. All 44 studies, including 54 comparisons, were eligible for meta-analysis. Thirty-seven studies [31, 41–76] encompassing 47 comparisons were eligible for meta-regression. Figure 1 presents the systematic search flow and the excluded studies with the reasons for exclusion.

### Study characteristics

Forty-four studies conducted among 2782 older adults were included in the meta-analysis. All included studies were published in English, with none published in Chinese. Eighteen studies (23 comparisons) measured dynamic balance using the BBS [41–43, 54, 56–59, 62, 63, 65–71, 75], three studies (five comparisons) measured dynamic balance using the FAB scale [77–79], 21 studies measured functional mobility using the TUG [31, 41, 44–50, 52, 53, 55, 58, 60, 61, 64, 72–74, 76, 80], and six  (two comparisons) measured DT ability using the dTUG [44, 45, 48, 49, 51, 61], and six studies (seven comparisons) assessed fall frequency [31, 51, 52, 81–83]. Only 37% (*n* = 16) of the studies [41–43, 45, 46, 48, 49, 52, 55, 56, 63, 64, 74, 76, 77, 83] DT training to participants’ performance level, and 57% (*n* = 25) of the studies delivered instructor-supervised training [41, 42, 45–51, 56, 57, 59–62, 64, 69, 70, 74–79, 81]. A summary of the included studies is provided in Table 1. Motor–cognitive DT exercise (93.0%, *n* = 39) was the most common DT type. Table 1 presents a summary of the included studies categorised by the outcome measures of interest.

### Quality of the included studies

**Fig. 2:**
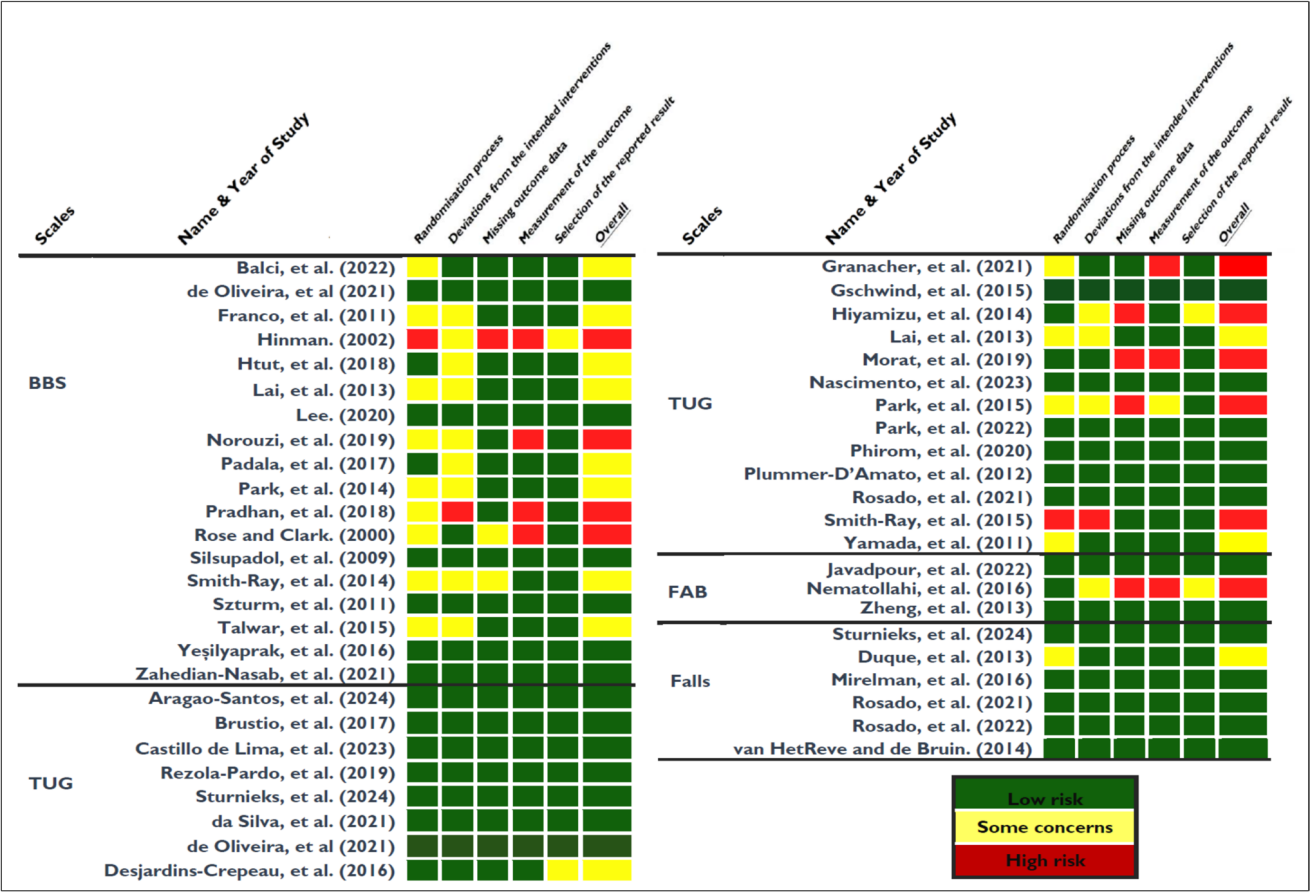
Risk of bias assessment of included studies for dynamic balance. *BBS* Berg balance scale, FAB Fullerton advanced balance, *TUG* Timed up and go

The mean PEDro score across all included studies was 6.05, indicating high methodological quality. Individual PEDro scores are reported in Table 2. Among the 44 studies, 70.5% (*n* = 31) had high PEDro scores, 25% (*n* = 11) had moderate PEDro scores and 4.5% (*n* = 2) had low PEDro scores (Table 1). Low risk of bias was found in 54% (*n* = 24) of the studies, while 25% (*n* = 11) of the studies had some concern for risk of bias (Fig. 2). The GRADE evidence quality ranged from low to moderate (Supplementary file 3). Evidence quality was very low for studies assessing dynamic balance using the BBS, low for studies assessing functional mobility using the TUG, moderate for studies assessing dynamic balance using the FAB scale and moderate for studies assessing fall frequency. Serious risk of bias and high heterogeneity were the primary reasons for downgraded evidence quality levels.

**Table 2:** Summary of included comparisons (54 comparisons in 44 studies) grouped by scales and falls

Variables/ characteristics		Balance (scales)				Falls
		BBS	TUG	dTUG	FAB	
Study design						
Number of studies (Intervention)		18(20)	21(24)	6(6)	3(4)	6(8)
Number of comparisons		23	24	7	5	7
Age, mean(SD)		73.51(4.24)	72.14(4.5)	70.4(3.72)	67.36(1.53)	76.23 (3.00)
PEDro score, mean(SD)		5.67(1.28)	6.38(1.17)	6.67(1.14)	6.33(1.5)	5.83(0.71)
PEDro score, range (minimum–maximum)		3–7	4–9	6–9	5–8	5–7
Sample size at randomization, mean(SD)		43(18.9)	70(109.17)	33(10.16)	69.33(20.82)	157.83(198.81)
Total sample size at randomization		780	1477	199	208	947
Sample size at randomization, Range (minimum–maximum)		18–88	17–514	16–44	44–95	30–514
Intervention duration(minutes), mean(SD)		42.34(18.55)	48.11(15.2)	57.5(14.75)	50(8.16)	47.39(22.19)
Intervention duration(minutes), range (minimum–maximum)		13–80	30–75	30–75	40–60	30–75
Pre-post intervention (weeks), mean(SD)		6.72(4.34)	13.19(6.44)	14(5.05)	6(1.63)	19.33 (7.08)
Pre-post intervention (weeks), Range (minimum–maximum)		3–24	4–24	6–24	4–8	6–26
Follow-up (weeks), mean(SD)		1.34(4.3)	2.29(5.27)	4(6.41)	–	10.5(12.53)
Follow-up (weeks), Range (minimum–maximum)		0–12	0–12	0–12	–	0–39
Control type	Active, comparisons/study number	19/14	13/11	2/2	5/3	4/3
	Usual care, comparisons/study number	4/4	11/10	5/4	–	3/3
Intensity adjusted to participants' ability, comparisons/study number		8/5	12/10	4/3	1/1	1/1
Exercise supervised, comparisons/study number		13/9	15/13	6/5	4/2	3/3
Minutes of exercise per week	90	8/5	3/3	–	–	–
	150	–	1/1	–	2/1	–
Days per week	3	16/12	12/11	3/3	5/3	5/4
Total intervention week	4	7/9	1/1	–	2/1	–
	13	–	1/1	–	–	–
Intensity	Mild (1.6–2.9 METs), comparisons/study number	1/1	3/3	1/1	–	2/2
	Moderate (3.0–5.9 METs), comparisons/study number	16/12	16/13	5/4	5/3	4/3
	High (≥ 6.0 METs) comparisons/study number	6/5	5/5	1/1	–	1/1
Challenge	Mild, comparisons/study number	8/6	6/5	1/1	1/1	2/2
	Moderate, comparisons/study number	12/9	16/14	5/4	4/2	5/4
	High, comparisons/study number	3/3	2/2	1/1	–	–
Adherence	≥ 95%, comparisons/study number	17/13	15/12	4/3	5/3	3/2
	≥ 95%, mean (SD)	99.54 (1.09)	99.79(0.67)	99.16(1.44)	91(9.91)	98.5(2.12)
	≥ 90%, comparisons/study number	21/16	19/16	6/5	5/3	5/4
	≥ 90%, mean (SD)	98.19(3.24)	97.92 (3.51)	96.5(3.5)	91(9.91)	95.5(4.14)

### Meta-analysis: effects of DT training on balance and falls

**Fig. 3:**
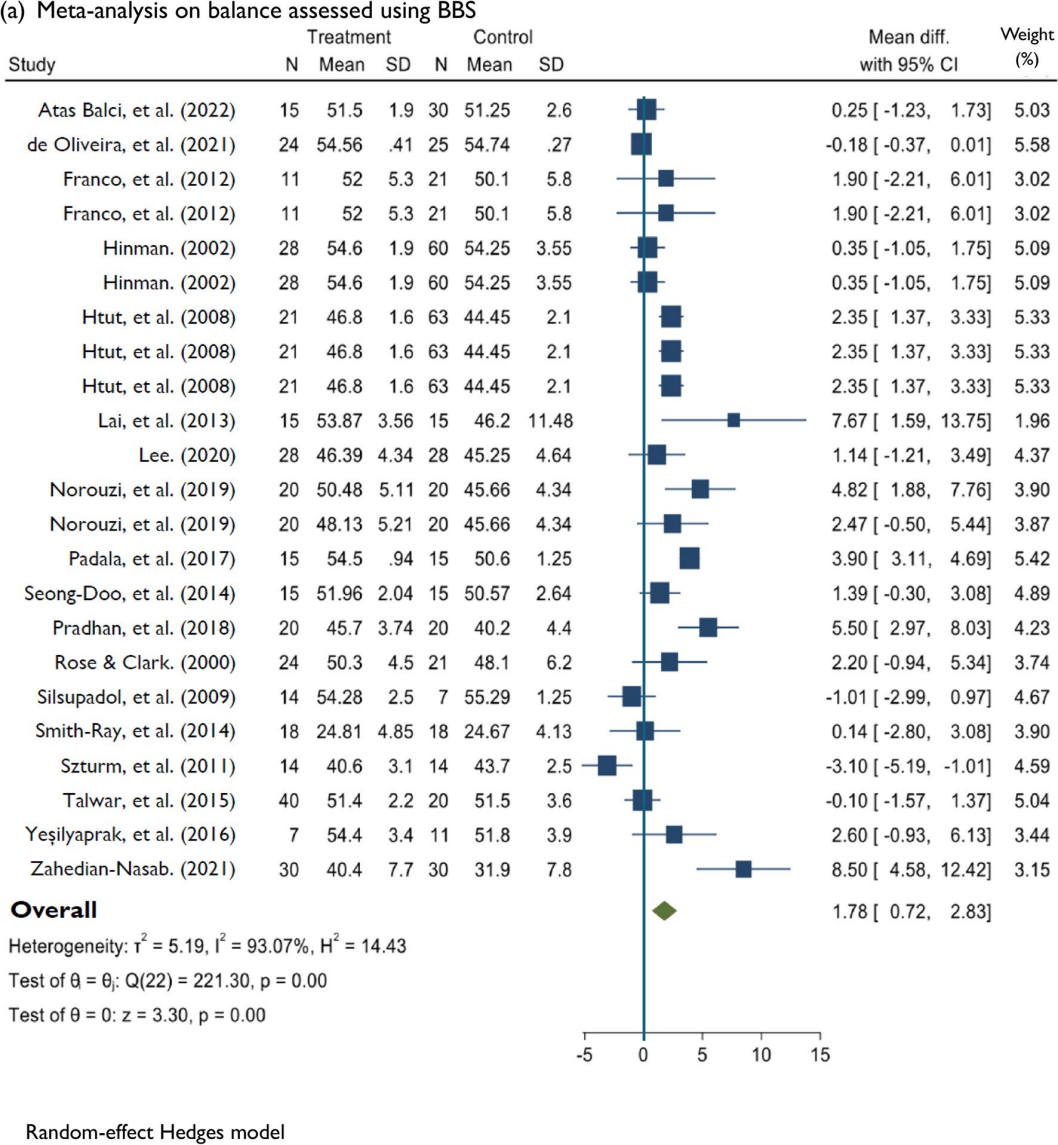

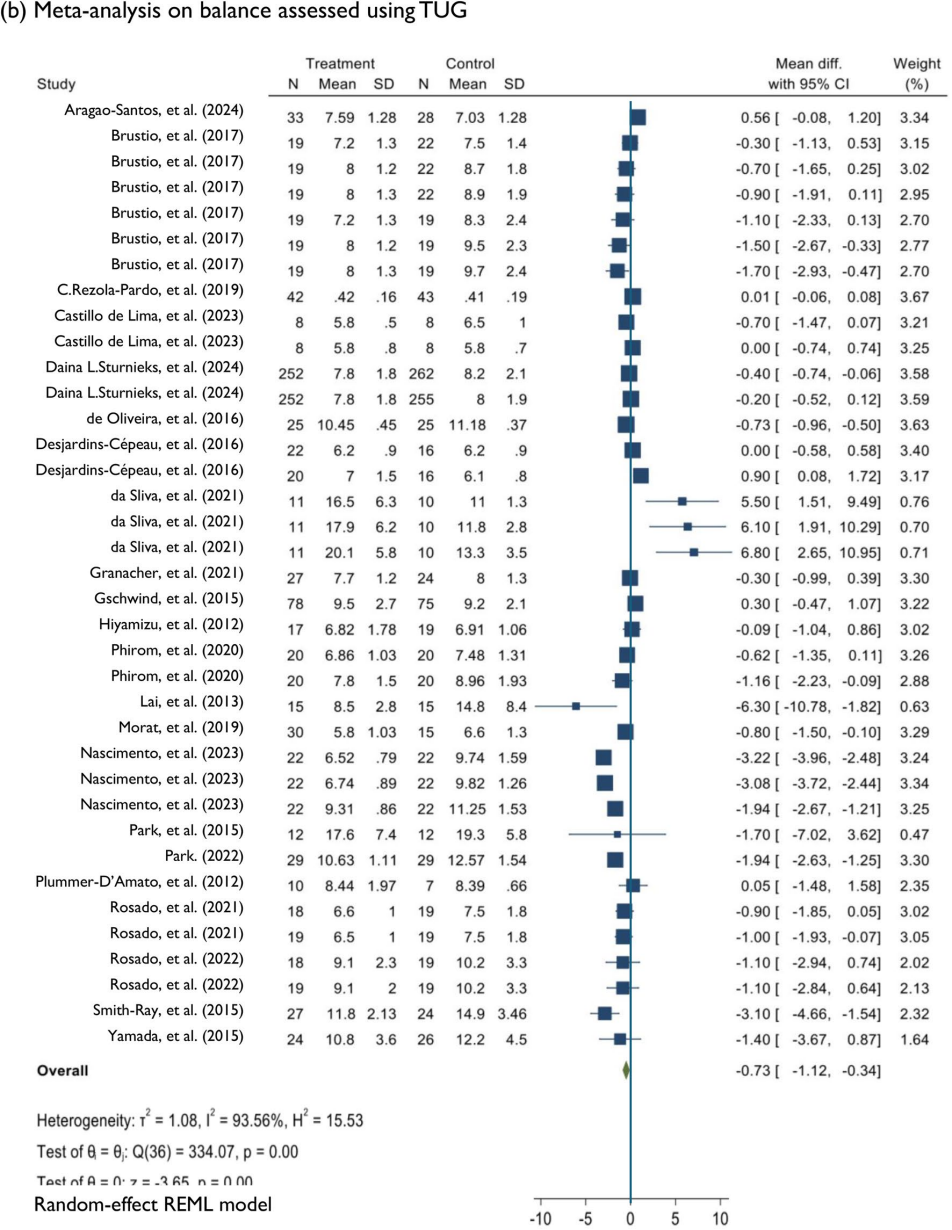

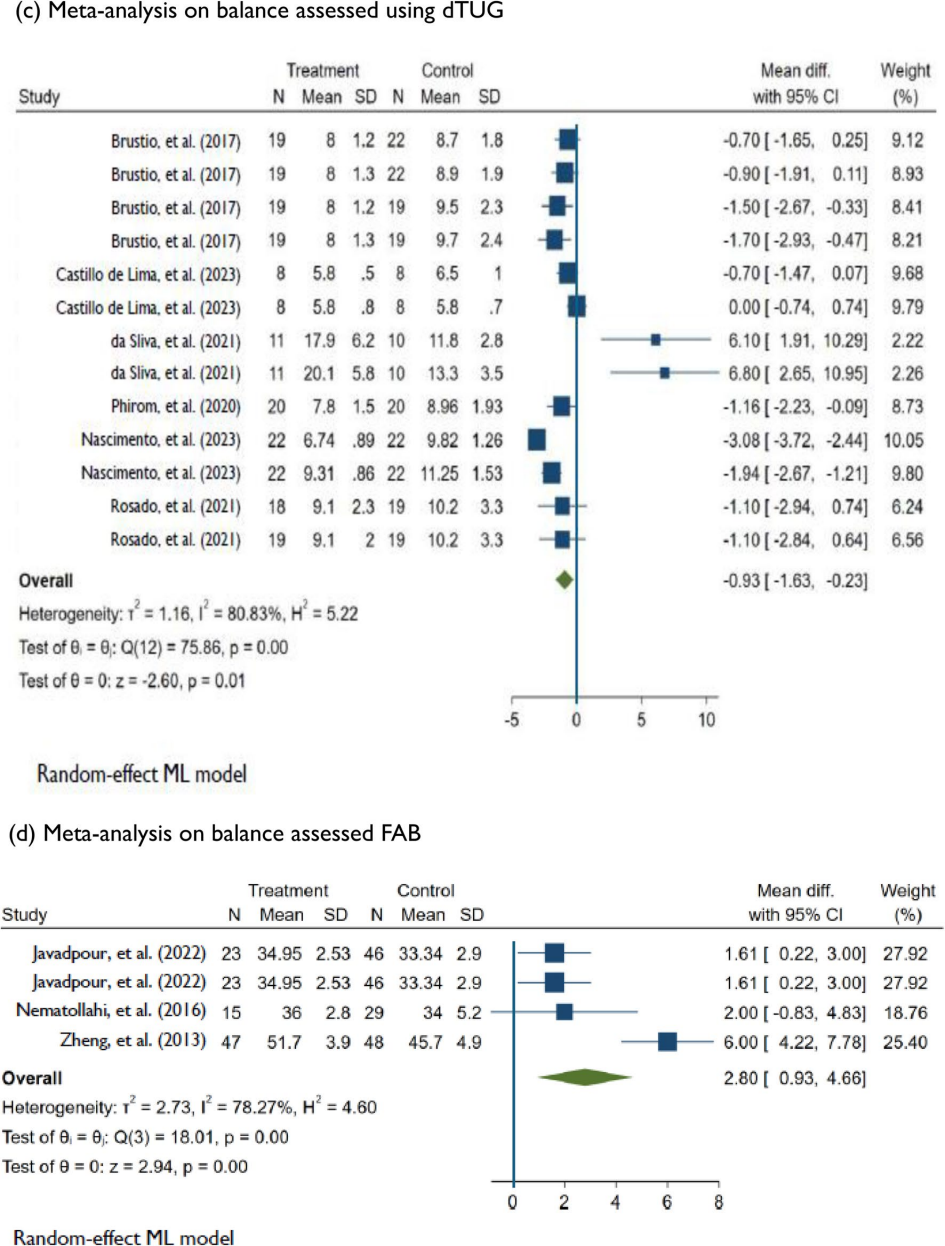

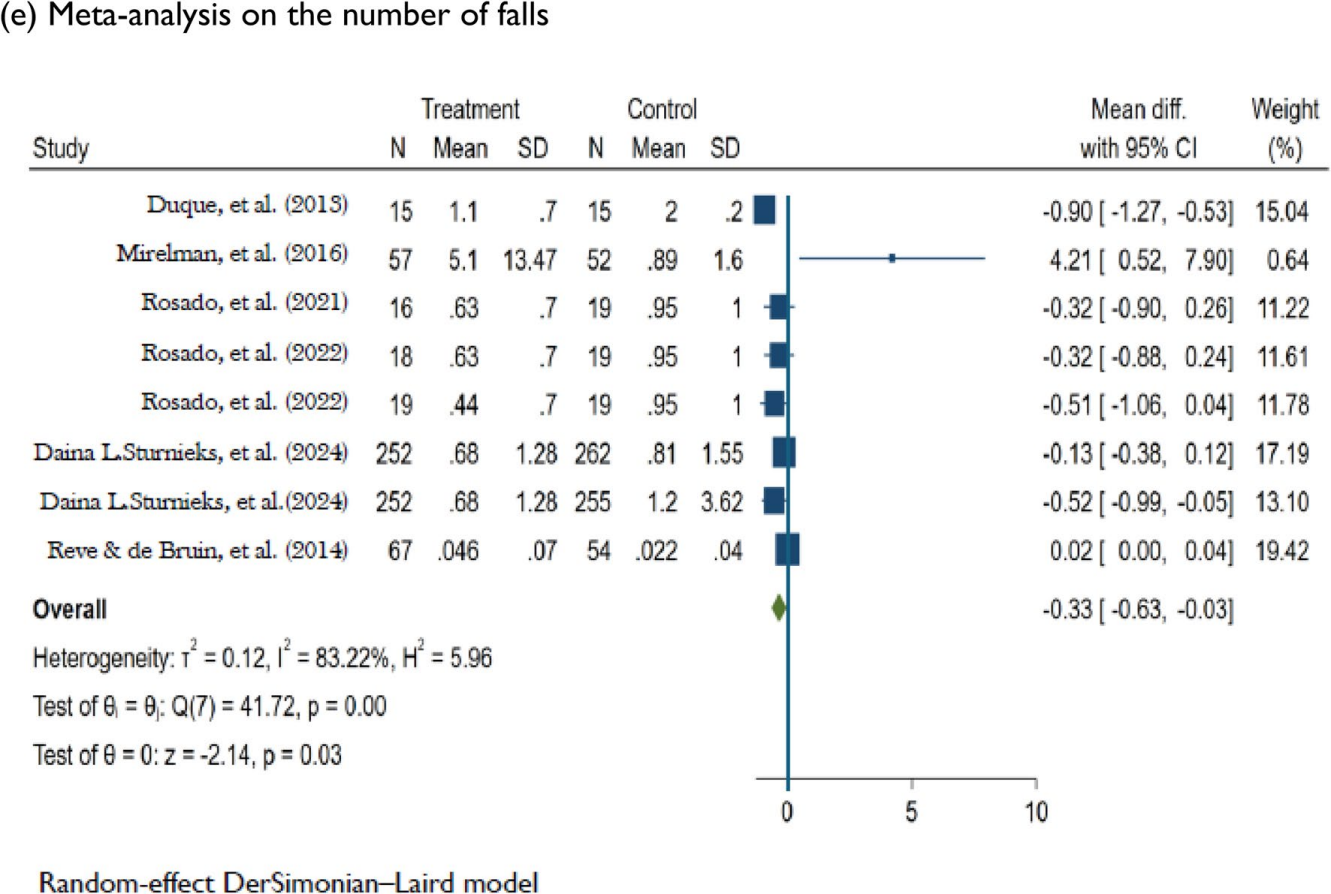
Random effects of DT training on improving balance measuring BBS, TUG, dTUG, and FAB, and reducing falls. *DT* Dua-task, *BBS* Berg Balance Scale, *TUG* Timed up and go, *dTUG* Dual-task TUG, *FAB* Fullerton advanced balance

Compared to control conditions, DT training had a significant effect on dynamic balance assessed using the BBS. This comparison was derived from 18 studies [41–43, 54, 56–59, 62, 63, 65–71, 75] of low to high risk of bias and very low evidence quality (MD = 1.78; 95%CI: 0.72, 2.83; *P* < 0.001; *I*^2^ = 93.07%; *n* = 18). Changes in functional mobility assessed using the TUG were significantly different between the DT and control groups (MD =  − 0.73; 95%CI: − 1.12, − 0.34; *P* < 0.001; *I*^2^ = 93.56%; *n* = 21). The comparison was based on 21 studies [31, 41, 44–53, 55, 58, 60, 61, 64, 72–74, 76] of low to high risk of bias and low evidence quality. Among six studies of low to high risk of bias [44, 45, 48, 49, 51, 61], DT training significantly improved DT ability assessed using the dTUG (MD =  − 0.93; 95%CI: − 1.63, − 0.23; *P* < 0.001; *I*^2^ = 80.83%; *n* = 6). DT training had a significant effect on dynamic balance measured using the FAB scale according to three studies [77–79] of low to high risk of bias and moderate evidence quality (MD = 2.80; 95%CI: 0.93, 4.66; *P* < 0.001; *I*^2^ = 78.27%, *n* = 3). Among the six studies assessing falls [31, 51, 80–83], which had low to some concerns for risk of bias and moderate evidence quality, DT training had a significant effect (MD =  − 0.33; 95%CI: − 0.63, − 0.03; *P* < 0.001; *I*^2^ = 83.22%; *n* = 6) on reducing fall frequency (Figs. 2 and 3).

### Subgroup analysis: effect of subgroup analysis of DT training on balance and falls

Subgroup analyses revealed a significant treatment effect of the DT intervention on dynamic balance assessed using the BBS among studies with higher PEDro-rated methodological quality (MD = 1.75; 95%CI: 1.20, 1.94; *P* < 0.001, *I*^2^ = 23%; *n* = 11 [41–43, 54, 56, 59, 62, 63, 68, 71]), a sample size ≥ 30 (MD = 1.70; 95%CI: 1.25, 2.14; *P* < 0.001; *I*^2^ = 47%; *n* = 15 [41–43, 54, 56–59, 62, 63, 65–67, 71, 75]), physiotherapist-guided training (MD = 1.44; 95%CI: 1.05, 1.83; *P* < 0.001; *I*^2^ = 23.82%; *n* = 7 [41, 42, 56, 59, 62, 63, 70]), motor–cognitive DT training (MD = 1.55; 95% CI: 1.09, 2.01; *P* < 0.001; *I*^2^ = 49%;* n* = 16 [41–43, 54, 56–59, 62, 63, 65, 66, 68–71]) and tmDT training (MD = 2.19; 95%CI: 1.81, 2.57; *P* < 0.001; *I*^2^ = 0.00%; *n* = 12 [42, 43, 56–59, 63, 65, 67, 69–71]). Studies with higher methodological quality based on a PEDro score ≥ 6 (MD =  − 0.77; 95%CI: − 0.96, − 0.59; *P* < 0.001; *I*^2^ = 59.06%; *n* = 17 [31, 41, 44, 45, 47–53, 55, 61, 72–74, 76]), a sample size ≥ 30 (MD =  − 0.53; 95%CI: −  0.64, − 0.42; *P* < 0.001; *I*^2^ = 13.73%; *n* = 17 [31, 41, 46–53, 55, 58, 60, 61, 72, 73, 76]), physiotherapist-guided training (MD =  − 2.33; 95%CI: − 2.7, − 1.96; *P* < 0.001; *I*^2^ = 0.00%; *n* = 3 [47, 50, 61]), motor–cognitive DT training (MD =  − 0.63; 95%CI: − 0.80, − 0.46; *P* < 0.001; *I*^2^ = 49%; *n* = 20 [31, 41, 44–48, 50–53, 55, 58, 60, 61, 64, 72–74, 76]) and conventional DT exercises (MD =  − 0.73; 95%CI: − 0.92, − 0.55; *P* < 0.001; *I*^2^ = 47.0%; *n* = 14 [41, 44–47, 49–51, 53, 55, 61, 72, 74, 76]) had a significant treatment effect on functional mobility assessed using the TUG. A non-significant intervention effect on dynamic balance based on the BBS was found for motor–motor (MD =  − 0.22.07; 95%CI: − 23.26, − 20.89; *P* < 0.001; *I*^2^ = 0.00%; *n* = 2 [67, 75]) and cognitive–cognitive (MD =  − 0.14; 95%CI: − 2.80, − 3.08; *P* = 0.93; *n* = 1 [43]) DT interventions. Likewise, a non-significant intervention effect on functional mobility per the TUG was found for motor–motor (MD =  − 0.74; 95%CI: − 1.10, − 0.38; *P* = 0.31; *I*^2^ = 0.00%; *n* = 1 [49]) and cognitive–cognitive (MD =  − 3.10; 95%CI: − 4.66, − 1.54; *P* < 0.001; *n* = 1 [52]) DT interventions. Studies with high methodological quality based on PEDro score ≥ 6 (MD =  − 0.25; 95%CI: − 0.42, − 0.07; *P* = 0.01; *I*^2^ = 0.00%; *n* = 4 [31, 51, 80, 82]), physiotherapist-guided training (MD =  − 0.73; 95%CI: − 1.05, − 0.42; *P* < 0.001; *I*^2^ = 0.00%; *n* = 2 [51, 81]) and conventional DT training (MD =  − 0.39; 95%CI: − 0.71, − 0.06; *P* = 0.02, *I*^2^ = 0.00%; *n* = 2 [51, 80]) had a significant treatment effect on fall frequency (Table 3).

**Table 3:** Effects of dual-task exercise on improving dynamic balance and functional mobility and reducing falls among older adults, results of the primary meta-analysis and subgroup analysis

Variables	BBS	TUG	FAB	Fall
All comparisons	MD (95%): 1.78* (0.72, 2.83)* *P*-value: < *0.001* I^2^: 93.07% N. study: 18 Participant: 1107	MD (95%): − 0.73 (− 1.12, − 0.34) *P*-value: < *0.001* I^2^: 93.56% N. study: 21 Participant: 2798	MD (95%): 2.80 (0.93, 4.66) *P*-value: < *0.001* I^2^: 78.27% N. study: 3 Participant: 277	MD (95%): − 0.30 (− 0.54, − 0.06) *P*-value: < *0.001* I^2^: 71.77% N. study: 6 Participant: 1570
Participants, n ≥ 30	MD (95%): *1.70 (1.25, 2.14)* *P*-value: < *0.001* I^2^: 47% N. study: 15 Participant: 1040	MD (95%): − *0.53 (− 0.64, -0.42)* *P*-value: < *0.001* I^2^:* 13.73%* N. study: 17 Participant: 2662		
Participants, n < 30	MD (95%): − 1.26 (− 2.75, 0.23) *P*-value: *0.02* I^2^: 17% N. study: 3 Participant: 67	MD (95%): 0.47 (− 0.39, 1.34) *P*-value: 28 I^2^: 42% N. study: 4 Participant: 136		
PEDro score ≥ 6	MD (95%): *1.75 (1.20, 1.94)* *P*-value: < *0.001* I^2^: 23*%* N. study: 11 Participant: 720	MD (95%): − 0.77 (− 0.96, − 0.59) *P*-value: < *0.001* I^2^: 59.06% N. study: 17 Participant: 2617	MD (95%):2.82 (1.62, 4.02) *P*-value: < *0.001* I^2^: 47% N. study: 2 Participant: 233	MD (95%): − *0.25 (− 0.42, − 0.07)* *P*-value: *0.01* I^2^:* 0.00%* N. study: 4 Participant: 1318
PEDro score < 6	MD (95%): 0.41 (-049, 1.31) *P*-value & I^2^: < *0.001* I^2^: 37% N. study: 4 Participant: 387	MD (95%): − 0.15 (− 0.78, 0.48) *P*-value: < 0.001 I^2^: 45% N. study: 4 & Participant: 181	MD (95%): 2.00 (-0.83, 4.83) *P*-value: < *0.17* I^2^: 0.00% N. study: 1 participant& 44	MD (95%): 0.02 (0.00, 0.04) *P*-value: < *0.001* I^2^: 0.00% N. study: 2 Participant: 252
Instructor	MD (95%): 1.40 (1.02, 1.77), *P*-value: < *0.001* I^2^: 21.75% N. study: 9 Participant: 593	MD (95%): − 0.90 (− 1.12, − 0.69) *P*-value: < *0.001* I^2^: 49% N. study:13 Participant: 1085		MD (95%): − *0.61 (− 0.86, − 0.37)* *P*-value: < *0.001* I^2^:* 0.00%* N. study: 3 Participant: 1312
Physiotherapist	MD (95%): 1.44 (1.05, 1.83) *P*-value: < *0.001* I^2^: 23.82% N. study: 7 Participant: 527	MD (95%): − 2.33 (− 2.7, − 1.96) *P*-value: < 0.001 I^2^: 0.00% N. study: 3 Participant: 233		MD (95%): − *0.73 (− 1.05, − 0.42)* *P*-value: < *0.001* I^2^:* 0.00%* N. study: 1 Participant: 70
Active control	MD (95%): 1.30 (0.83, 1.77) *P*-value: < *0.001* I^2^: 47% N. study: 14 Participant: 887	MD (95%): − 0.28 (− 0.41, − 0.15) *P*-value: < *0.001* I^2^: 15.26% N. study: 11 Participant: 1094	MD (95%): 3.11 (2.08, 4.13) *P*-value: < *0.001* I^2^: 0.00% N. study: 3 Participant: 277	MD (95%): − 0.03 (− 0.19, 0.13) *P*-value: 0.05 I^2^: 33.15% N. study: 3 Participant: 805
Usual care/ no intervention	MD (95%): 2.8 (1.07, 4.53) *P*-value: 0.11 I^2^: 26.57% N. study: 4 Participant: 220	MD (95%): − 1.07 (− 1.32, − 0.81) *P*-value: < *0.001* I^2^: 37% N. study: 10 Participant: 1654		MD (95%): − 0.61 (− 0.89, − 0.34) *P*-value: 0.3 I^2^: 25.73% N. study: 3 Participant: 765
tmDT	MD (95%): 2.19 (1.81, 2.57) *P*-value: < *0.001* I^2^: 0.00% N. study: 12 Participant: 792	MD (95%): − 0.55 (− 0.88, − 0.22) *P*-value: < *0.001* I^2^: 43% N. study: 7 Participant: 1674		MD (95%): − 0.17 (− 0.31, − 0.02) *P*-value: < *0.001* I^2^: 35.08% N. study: 4 Participant: 1382
Conventional DT	MD (95%): 1.01 (-0.02, 2.04) *P*-value: < 0.001 I^2^: 67% N. study: 6 Participant: 315	MD (95%): − *0.73 (− 0.92, − 0.55)* *P*-value: < *0.001* I^2^: 47.0% N. study: 14 Participant: 1124		MD (95%): − 0.39 (− 0.71, − 0.06) *P*-value: 0.02 I^2^: 0.00% N. study: 2 Participant: 188
Motor-cognitive	MD (95%):*1.55 (1.09, 2.01)* *P*-value: < *0.001* I^2^: 49% N. study: 16 Participant: 1002	MD (95%): − *0.63 (− 0.80, − 0.46)* *P*-value: < *0.001* I^2^: 49% N. study: 20 Participant: 2561		
Motor-motor	MD (95%): − 22.07 (− 23.26, − 20.89) *P*-value: < 0.001 I^2^: 0.00% N. study: 2 Participant: 105	MD (95%): − 0.74 (− 1.10, − 0.38) *P*-value: 0.31 I^2^: 0.00% N. study: 1 Participant: 237		
Cognitive-cognitive	MD (95%): 0.14 (− 2.80, − 3.08) *P*-value: 0.93 I^2^: 0.001% N. study: 1 Participant: 36	MD (95%): − 3.10 (− 4.66, − 1.54) *P*-value: < 0.001 I^2^: 0.001% N. study: 1 Participant: 51		

Statistically and positively significant comparisons are shown in italics, *N* Number

*BBS* Berg balance scale, *N* Number, *tmDT* Technology-mediated dual-task, *TUG* Time up and go, *FAB* Fullerton advanced balance, > Greater than, < Less than, ≥ Greater than equal, ≤ Less than equal

### Meta-regression: effects of study features of DT training on dynamic balance and functional mobility

The effects of DT training study features on balance are shown in Table 4. Among the studies assessing dynamic balance using the BBS, studies that delivered interventions for 90 min minutes per week (meta-regression coefficient = 0.83; 95%CI: 0.45, 1.21; *P* < 0.001; *I*^2^ = 9.05%; *n* = 5 [42, 54, 58, 65, 67]), three days per week, (0.64; 95%CI: 0.42, 0.86; *P* < 0.001; *I*^2^ = 2.62%; *n* = 12 [41, 42, 54, 57, 58, 62, 63, 65, 66, 68, 70, 75]), 30 min per session for four weeks (0.75; 95%CI: 0.15, 1.36; *P* = 0.015; *I*^2^ = 1.46%; *n* = 7 [54, 57, 59, 62, 66, 68, 75]), at a moderate challenge level (0.59; 95%CI: 0.36, 0.82; *P* < 0.00; *I*^2^ = 2.39%; *n* = 9 [41–43, 54, 63, 65, 67, 69, 71]), at a moderate intensity (0.32; 95%CI: 0.004, 0.63; *P* = 0.05; *I*^2^ = 9.26%; *n* = 12 [42, 56–59, 62, 63, 66–70]) and with 95% adherence (0.71; 95%CI: 0.49, 0.94; *P* < 0.001; *I*^2^ = 2.47%; *n* = 13 [41, 42, 54, 56, 58, 62, 63, 65–67, 70, 71, 75]) were highly significant. Among the studies assessing functional mobility using the TUG, studies that delivered interventions for 150 min minutes per week (0.89; 95%CI: 0.02, 1.76; *P* < 0.04; *I*^2^ = 7.73%; *n* = 1 [55]), three days per week (0.90; 95%CI: 0.03, 1.77; *P* < 0.04; *I*^2^ = 89%; *n* = 11 [41, 45, 46, 48, 51, 52, 55, 58, 60, 64, 73], 50 min per session for 13 weeks (0.89; 95%CI: 0.02, 1.76; *P* = 0.04; I^2^ = 7.73%; *n* = 1 [76]), at a mild challenge level (0.92; 95%CI: 0.19, 1.66; *P* < 0.01; *I*^2^ = 0.00%; *n* = 5 [46, 51, 53, 58, 60]), at a moderate intensity (0.56; 95%CI: 0.16, 0.95; *P* = 0.006; *I*^2^ = 9%; *n* = 13 [31, 44–46, 48–50, 55, 58, 60, 72–74]) and with 95% adherence (0.65; 95%CI: 0.09, 1.2; *P* < 0.02; *I*^2^ = 84%; *n* = 12 [31, 41, 44, 46, 48–50, 58, 64, 72, 74, 76]) were highly significant.

**Table 4:** Results of meta-regression explore effect of study-level characteristics on improvement of dynamic balance and functional mobility in older adults

Variables		BBS	TUG	Interpretation
Study design				
PEDro score		Coef. (95%): 0.13 (− 0.61, 0.88) *P*-value: 0.15 I^2^: 90% N. study: 18 Participant: 1107	Coef. (95%): − 0.103 (− 0.15, − 0.05) *P*-value: 0.00 I^2^: 92% N. study: 21 Participant: 2798	Instructors have a significant effect on dual-task intervention in improving dynamic balance Compared with instructors performing a dual-task intervention with physiotherapist instructions, this significantly improves functional mobility Dual-task exercise tailored to the ability of the performance level of older adults significantly improves dynamic balance and functional mobility
Sample Size		Coef. (95%): − 0.006 (− 0.06, 0.05) *P*-value: 0.83 I^2^: 93% N. study: 18 Participant: 1107	Coef. (95%): − 0.002 (− 0.005, 0.001) *P*-value: 0.23 I^2^: 97% N. study: 21 Participant: 2798	
Instructor		Coef. (95%): *0.31 (0.77, 0.85)* *P*-value: *0.00* I^2^:* 2.15%* N. study: 9 Participant: 593	Coef. (95%): -0.79* (-1.25, -0.33)* *P*-value: *0.001* I^2^: 92.49*%* N. study: 13 Participant: 1085	
Physiotherapy instructor		Coef. (95%): *0.29 (0.12, 0.47)* *P*-value: *0.001* I^2^:* 0.00%* N. study: 7 Participant: 527	Coef. (95%): 0.97 (1.51, 0.47) *P*-value: 0.001 I^2^: 0.00% N. study: 3 Participant: 233	
Exercise tailored to ability		Coef. (95%): *0.61 (0.37, 0.85)* *P*-value: < *0.001* I^2^:* 2.15%* N. study: 5 Participant: 398	Coef. (95%): 0.65 (0.3, 1.0) *P*-value: < *0.001* I^2^: 51.87*%* N. study: 10 Participant: 746	
Sample features				
Average age		Coef. (95%): 0.02 (0.01, 0.04) *P*-value: *0.001* I^2^: 91.5% N. study: 18 Participant: 1107	Coef. (95%): 0.05 (0.015, 0.08) *P*-value: 0.005 I^2^: 43.48% N. study: 21 Participant: 2798	Dual-task intervention improves the dynamic balance and functional mobility of older adults
Intervention features				
Minutes of exercise per week	90	Coef. (95%): *0.83 (0.45, 1.21)* *P*-value: < *0.001* I^2^:* 9.05%* N. study: 5 Participant: 360		Ninety minutes of DT exercise is required weekly to improve dynamic balance
	150		Coef. (95%): *0.89* (0.02, 1.76) *P*-value: 0.04 I^2^: 7.73% N. study: 1 Participant: 61	150 min of DT exercise is required weekly to improve functional mobility
Days per week	3	Coef. (95%): 0.64* (0.42, 0.86)* *P*-value: < *0.001* I^2^:* 2.62%* N. study: 12 Participant: 555	Coef. (95%): *0.90 (0.03, 1.77)* *P*-value: *0.04* I^2^: *89.58%* N. study: 11 Participant: 1324	Three days are required to practice DT to have an expected improvement in balance
Total intervention week	4	Coef. (95%): 0.75 (0.15, 1.36) *P*-valueI^2^: 0.015 I^2^: 1.46% N. study: 7 Participant: 498		DT intervention requires implementation for 4 weeks and 13 weeks to achieve significant improvement in dynamic balance and functional mobility, respectively
	13		Coef. (95%): *0.89* (0.02, 1.76) *P*-value:* 0.04* I^2^: 7.73% N. study: 1 Participant: 85	
Intensity	Moderate (3.0–5.9 METs)	Coef. (95%): *0.32 (0.004, 0.63)* *P*-value: *0.05* I^2^:* 9.26%* N. study: 12 Participant: 818	Coef. (95%): *0.56 (0.16, 0.95)* *P*-value: *0.006* I^2^: 9% N. study: 13 Participant: 2192	DT intervention with moderate intensity requires improving balance
Challenge	Mild		Coef. (95%): *0.92 (0.19, 1.66)* *P*-value:* 0.01* I^2^: 0.00*%* N. study: 5 Participant: 369	DT intervention with mild and moderate challenges requires improving dynamic balance and functional mobility
	*Moderate*	Coef. (95%): *0.59 (0.36, 0.82)* *P*-value: < 0.001 I^2^: 2.39% N. study: 9 Participant: 542		
Adherence	≥ *95%*	Coef. (95%): *0.71 (0.49, 0.94)* *P*-value: < *0.00* I^2^: 2.47*%* N. study: 13 Participant: 781	Coef. *(95%): 0.65 (0.09, 1.2*) *P*-value: *0.02* I^2^:* 84.68%* N. study: 12 Participant: 2023	Older adults require 95% adherence to DT intervention to improve balance

Statistically and positively significant comparisons are shown in italics

*BBS* Berg balance scale, *DT* Dual task, *METs* Metabolic equivalents, *TUG* Time up and go, *FAB* Fullerton advanced balance, > Greater than, < Less than, ≥ Greater than equal, *PEDro* Physiotherapy evidence database, *N* Number

### Consequences of small-study effect

Significant asymmetry was not present in the funnel plots, indicating no small-study effects (Supplementary file 4). We considered studies with sample sizes smaller than 30 in the examination of small-study effects. Ten studies using the BBS and TUG had sample sizes under 30. Egger’s test suggested no small-study effect in the meta-analysis of studies assessing dynamic balance using the BBS (β(SE) = 5.84(3.34), *P* = 0.08) and functional mobility using the TUG (β(SE) = 2.08(0.49), *P* = 0.22).

## Discussion

This systematic review with meta-analysis and meta-regression provides very low to moderate quality evidence that DT training improves dynamic balance and functional mobility and reduces falls among older adults. This is the first study to use meta-regression to examine if intensity, challenge, adherence and training hours per week are linked to greater benefits following DT interventions in this population. We found that DT training for 90 min spread over three days per week (30 min per session) for four weeks, performed at moderate intensity and challenge levels, with at least 95% adherence, elicited the best dynamic balance outcomes.

Meta-analysis revealed an MD of 1.78 for the BBS and − 0.73 for the TUG. Though the comparisons were significant, the MD did not meet the MCID threshold of 3 for the BBS [84] and − 2.2 for the TUG [85]. As none of the studies in this meta-analysis met the optimal treatment dosage criteria, we hypothesise a higher MD if the optimal treatment dosage is met. In addition, only 33% of the studies included in the meta-analysis of dynamic balance assessed using the BBS had a low risk of bias, and the quality of the evidence was very low. Recent reviews analysing dynamic balance and functional mobility in healthy older adults [11, 18]. The reviews found smaller effect sizes for the BBS and TUG than those found in our study, suggesting that the effects were statistically significant but trivial compared with the MCID threshold value [86]. The previous review’s meta-analysis included studies with low to high methodological quality [86]. The studies included in our meta-analysis for the BBS and TUG were generally of good methodological quality, with a range of low to high risk of bias and very low to low evidence quality. Future high-quality studies evaluating DT training at the optimal treatment dosage are thus recommended.

Our meta-analysis reported DT training significantly reduced fall frequency among healthy older adults residing in community, residential care and nursing homes. This finding aligns with a recent review, which reported that DT training reduced the incidence of falls among community-dwelling older adults [11]. Despite these promising results, a limitation across included studies is the method of data collection. All six included studies retrospectively collected fall on self-reported falls using a questionnaire at baseline assessment [31, 51, 80–83]. At post-assessment, three studies prospectively collected data on self-reported falls using fall diaries [31, 82, 83] while three did not use fall diaries [51, 80, 81]. The self-reported approach is subject to recall bias and may potentially result in underreporting or overreporting fall data [30, 33]. These limitations impact the accuracy of fall incidence data and lead to underestimation of intervention effects [87]. Future studies should adopt more rigorous, prospective fall monitoring approaches, like daily diaries, routine phone check-ins, or wearable sensors, to improve data precision and comparability.

In our analysis, DT interventions improved DT ability. Risk of bias was low among all studies included in this comparison. DT ability improves secondary to enhanced executive functioning [88], as explained by executive function theory [9]. The process involves enhancing task inhibition, updating working memory and shifting between tasks to improve balance while promoting cognitive function [9]. Through DT training, the primary task, either motor or cognitive, is prioritised, working memory is updated and an effective transition between motor and cognitive tasks is facilitated [9]. These mechanisms improve executive function and in turn improve DT ability in older adults.

Motor–cognitive DT interventions improved dynamic balance according to our analysis. Few studies utilised motor–motor interventions, and subgroup analysis revealed non-significant effects of motor–motor training on dynamic balance using the BBS and functional mobility using the TUG. Likewise, cognitive–cognitive DT interventions had a non-significant effect on dynamic balance assessed using the BBS, though were significant in improving functional mobility assessed using the TUG. The available recent literature does not support the use of motor–motor and cognitive–cognitive exercises for improving dynamic balance and functional mobility and reducing falls [11, 18], and it is notable that the number of studies investigating motor–motor (*n* = 3) and cognitive–cognitive (*n* = 2) DT training was low in our review. Additional studies comparing the treatment effects of different DT training types on balance and falls in this population are recommended.

DT interventions are widely used to reduce balance deficits and prevent falls in diverse populations of older adults. The use of motor–cognitive DT interventions has been reported in Asia [42], South America [41], North America [74], Europe [60], and Australia [73], and in middle-income [78] and high-income [74] countries. Fewer reports from India, Germany and the USA have assessed motor–motor DT training [67, 72, 75], while cognitive–cognitive DT interventions are even rarer [43, 52], which may be attributed to an inaccessibility of technology and a lack of evidence.

This study included older adults of both sexes, though participation was higher among females than males. A previous review reported similar findings [7]. Sex differences have been found in the incidence of balance deficits and risk of falls among older adults, with females having a higher incidence of both compared to men [89, 90]. Sex differences DT training treatment effects may additionally exist [91], though the influence of sex on DT interventions is uncertain due to a lack of sex-stratified analysis in previous study [92]. Future studies should thus include sex-stratified analysis to determine if DT intervention effects vary between sexes.

A previous review examining dose–response relationships in balance training found that a dosage of three sessions per week, each lasting 31–45 min, for 11–12 weeks was most effective for improving balance performance [93]. Another review revealed that exercise reduced falls in older adults by 21%, with greater benefits observed for high-challenge exercises performed for more than three hours per week [16]. However, these studies [16, 93] did not include optimal exercise intensity. Our meta-analysis results suggest an optimal dosage for improving balance and reducing falls. In contrast to previous evidence [93], our meta-regression confirmed that DT training three times per week for an average of 30 min each session for four weeks is optimal for improving dynamic balance in older adults.

Our study proposes a minimum adherence of 95% DT training of the proposed session is effective for improving balance. However, this level of adherence could pose challenges for feasibility and clinical practice. Achieving a 95% adherence rate requires older adults to attend nearly all sessions, a challenge in real-world clinical settings due to barriers such as health issues, transportation, or other commitments. An earlier study has highlighted that high adherence rates are often difficult to achieve outside of controlled research environments [94]. Therefore, while 95% adherence rate is required for balance improvement, clinicians and practitioners should consider individual patient circumstances and potential barriers to adherence when designing DT interventions.

Our findings should be interpreted with caution due to the following limitations. (1) Meta-analysis findings, with nearly 100% heterogeneity, indicate significant variability among the included studies, which restricts the interpretability and generalizability [95]. To address this limitation, we used a random-effects model and conducted subgroup to explore potential sources of variability. Despite high heterogeneity, our findings offer a broader understanding of the intervention’s effects. (2) Among the three DT training types, the number of motor–motor [67, 72, 75] and cognitive–cognitive DT training studies was low [43, 52]. Therefore, generalising the findings to all DT training types may not be appropriate. (3) The findings of our review are limited to the five outcome measures of interest. Other standardized measures of dynamic balance, such as the Mini-Balance Evaluation Systems Test (Mini-BESTest) were not considered. The findings based on such outcome measures are still unknown; therefore, future reviews may consider including those outcome measures. (4) Meta-regression analysis was not performed for the FAB scale or fall frequency due to the inadequate number of available studies. (5) Participants in the majority of included studies were ambulatory and independent, limiting the study’s generalisability to frail older adults. (6) As only studies in English and Chinese were considered, relevant studies in other languages may have been excluded. (7) We did not account for unpublished studies, which may have excluded relevant articles.

This review has several notable strengths. (1) This is the first systematic review with meta-analysis and meta-regression to compare the effects of DT training with single-task exercise or no intervention on balance and falls in older adults. Previous systematic reviews have comprised qualitative syntheses or meta-analyses without meta-regression [7, 15], (2) Comprehensive electronic database and manual searches were conducted to identify a broad range of relevant studies [96], and (3) Our use of PEDro, Cochrane risk of bias and GRADE recommendations increases the transparency of the review’s findings.

## Conclusion

There is very low to moderate quality evidence for the use of DT training as a single intervention to improve dynamic balance and functional mobility and reduce falls in older adults. We recommend DT interventions with moderate challenge and intensity, a minimum adherence of 95%, 30 min of training per session, a frequency of three days per week and a duration of four weeks to effectively improve dynamic balance. Functional mobility following DT training improved with mild challenge and moderate intensity, a minimum adherence of 95%, 50 min of training per session, a frequency of three days per week and a duration of 13 weeks. Additional high-quality studies are needed to determine the impact of DT training on falls and compare the treatment effects of different DT training types on balance and falls.

## Supplementary Information

Below is the link to the electronic supplementary material.

Supplementary file1 (DOCX 86 KB)

## Data Availability

Not applicable.
