# Supplementary material for: Effectiveness of dual-task exercise in improving balance and preventing falls among older adults: systematic review with meta-analysis and meta-regression
Source: Eur Geriatr Med. 2025 Oct 28;16(6):2047–83. doi: 10.1007/s41999-025-01328-3 (PMC12743702; doi:10.1007/s41999-025-01328-3)
Supplement: Supplementary file 1 — Supplementary file1 (DOCX 86 KB) [file 41999_2025_1328_MOESM1_ESM.docx]

**Supplementary file**

**Effectiveness of dual-task exercise in improving balance and preventing falls among older adults: Systematic review with meta-analysis and meta-regression**

**Supplementary file 1:** PRISMA checklist

| **Section and Topic** | **Item #** | **Checklist item** | **Location where item is reported** |
| --- | --- | --- | --- |
| **TITLE** | | |  |
| Title | 1 | Identify the report as a systematic review. | 1 |
| **ABSTRACT** | | |  |
| Abstract | 2 | Abstracts checklist. | 1 |
| **INTRODUCTION** | | |  |
| Rationale | 3 | Describe the rationale for the review in the context of existing knowledge. | 2 |
| Objectives | 4 | Provide an explicit statement of the objective(s) or question(s) the review addresses. | 2 |
| **METHODS** | | |  |
| Eligibility criteria | 5 | Specify the inclusion and exclusion criteria for the review and how studies were grouped for the syntheses. | 3 |
| Information sources | 6 | Specify all databases, registers, websites, organisations, reference lists and other sources searched or consulted to identify studies. Specify the date when each source was last searched or consulted. | 3 |
| Search strategy | 7 | Present the full search strategies for all databases, registers and websites, including any filters and limits used. | 3 |
| Selection process | 8 | Specify the methods used to decide whether a study met the inclusion criteria of the review, including how many reviewers screened each record and each report retrieved, whether they worked independently, and if applicable, details of automation tools used in the process. | 6 |
| Data collection process | 9 | Specify the methods used to collect data from reports, including how many reviewers collected data from each report, whether they worked independently, any processes for obtaining or confirming data from study investigators, and if applicable, details of automation tools used in the process. | 6 |
| Data items | 10a | List and define all outcomes for which data were sought. Specify whether all results that were compatible with each outcome domain in each study were sought (e.g. for all measures, time points, analyses), and if not, the methods used to decide which results to collect. | 4,7 |
|  | 10b | List and define all other variables for which data were sought (e.g. participant and intervention characteristics, funding sources). Describe any assumptions made about any missing or unclear information. | 4,7 |
| Study risk of bias assessment | 11 | Specify the methods used to assess risk of bias in the included studies, including details of the tool(s) used, how many reviewers assessed each study and whether they worked independently, and if applicable, details of automation tools used in the process. | 5 |
| Effect measures | 12 | Specify for each outcome the effect measure(s) (e.g. risk ratio, mean difference) used in the synthesis or presentation of results. | 8 |
| Synthesis methods | 13a | Describe the processes used to decide which studies were eligible for each synthesis (e.g. tabulating the study intervention characteristics and comparing against the planned groups for each synthesis (item #5)). | N/A |
|  | 13b | Describe any methods required to prepare the data for presentation or synthesis, such as handling of missing summary statistics, or data conversions. | 8 |
|  | 13c | Describe any methods used to tabulate or visually display results of individual studies and syntheses. | 8-10 |
|  | 13d | Describe any methods used to synthesize results and provide a rationale for the choice(s). If meta-analysis was performed, describe the model(s), method(s) to identify the presence and extent of statistical heterogeneity, and software package(s) used. | 8-10 |
|  | 13e | Describe any methods used to explore possible causes of heterogeneity among study results (e.g. subgroup analysis, meta-regression). | 8-10 |
|  | 13f | Describe any sensitivity analyses conducted to assess robustness of the synthesized results. | 8-10 |
| Reporting bias assessment | 14 | Describe any methods used to assess risk of bias due to missing results in a synthesis (arising from reporting biases). | 8-10 |
| Certainty assessment | 15 | Describe any methods used to assess certainty (or confidence) in the body of evidence for an outcome. | 8-10 |
| **RESULTS** | | |  |
| Study selection | 16a | Describe the results of the search and selection process, from the number of records identified in the search to the number of studies included in the review, ideally using a flow diagram. | 10 |
|  | 16b | Cite studies that might appear to meet the inclusion criteria, but which were excluded, and explain why they were excluded. | N/A |
| Study characteristics | 17 | Cite each included study and present its characteristics. | 11 |
| Risk of bias in studies | 18 | Present assessments of risk of bias for each included study. | 11 |
| Results of individual studies | 19 | For all outcomes, present, for each study: (a) summary statistics for each group (where appropriate) and (b) an effect estimate and its precision (e.g. confidence/credible interval), ideally using structured tables or plots. | 11 |
| Results of syntheses | 20a | For each synthesis, briefly summarise the characteristics and risk of bias among contributing studies. | 11 |
|  | 20b | Present results of all statistical syntheses conducted. If meta-analysis was done, present for each the summary estimate and its precision (e.g. confidence/credible interval) and measures of statistical heterogeneity. If comparing groups, describe the direction of the effect. | 12 |
|  | 20c | Present results of all investigations of possible causes of heterogeneity among study results. |  |
|  | 20d | Present results of all sensitivity analyses conducted to assess the robustness of the synthesized results. | 12 |
| Reporting biases | 21 | Present assessments of risk of bias due to missing results (arising from reporting biases) for each synthesis assessed. | 11 |
| Certainty of evidence | 22 | Present assessments of certainty (or confidence) in the body of evidence for each outcome assessed. | 12-13 |
| **DISCUSSION** | | |  |
| Discussion | 23a | Provide a general interpretation of the results in the context of other evidence. | 15 |
|  | 23b | Discuss any limitations of the evidence included in the review. | 18 |
|  | 23c | Discuss any limitations of the review processes used. | 18 |
|  | 23d | Discuss implications of the results for practice, policy, and future research. | 16-18 |
| **OTHER INFORMATION** | | |  |
| Registration and protocol | 24a | Provide registration information for the review, including register name and registration number, or state that the review was not registered. | 3 |
|  | 24b | Indicate where the review protocol can be accessed, or state that a protocol was not prepared. | 3 |
|  | 24c | Describe and explain any amendments to information provided at registration or in the protocol. | N/A |
| Support | 25 | Describe sources of financial or non-financial support for the review, and the role of the funders or sponsors in the review. | 20 |
| Competing interests | 26 | Declare any competing interests of review authors. | 20 |
| Availability of data, code and other materials | 27 | Report which of the following are publicly available and where they can be found: template data collection forms; data extracted from included studies; data used for all analyses; analytic code; any other materials used in the review. | N/A |

**Supplementary file 2**: Search strategy for PubMed

|  |  | **General Search Terms** |
| --- | --- | --- |
| **Themes** | **Subject Areas** | **Search Terms Used (Each Term within Criteria Combined with Boolean Operator ‘OR’)** |
| **Population** | Older  AND | ((older adult) OR (elderly) OR (senior) OR (older) OR (aged)) AND |
| **Intervention** | Dual—task AND | ((dual—task) OR (dual task) OR (cognitive motor interference) OR (psychomotor performance) OR (concurrent task) OR (dual tssask paradigm) OR (attention-demanding task) OR (divided attention)) AND |
| **Outcome** | Balance  OR | ((balance) OR (equilibrium) OR (posture) OR (postur*) OR (postural control) OR (postural stability) OR (postural balance) OR (postural sway) OR (sway) OR (center of pressure) OR (center of mass) OR (posture equilibrium)) OR |
|  | Fall  AND | ((fall) OR (falling) OR (falls) OR (falls, accidental) OR (fall, accidental) OR (slip and fall) OR (fall and slip)) AND |
| **Study** | Randomize control trial NOT | ((Randomized controlled trial) OR (Randomized allocation) OR (Random*) OR (Clinical trial)) NOT |
|  |  | ((Animal) OR (Systematic Review)) |

**Supplementary file 3**: The GRADE assessment for the quality of evidence among the included studies

| **Certainty assessment** | | | | | | | **№ of patients** | | **Effect** | **Certainty** |
| --- | --- | --- | --- | --- | --- | --- | --- | --- | --- | --- |
| **Outcome/ scales** | **№ of studies** | **Study design** | **Risk of bias** | **Inconsistency** | **Indirectness** | **Imprecision** | **Intervention** | **Control** | **MD(CI)**  **I^2^** |  |
| Balance/  BBS | 18 | RCT | Not serious^a^ | Serious^b^ | Not serious | Not serious | 460 | 660 | 1.78 (0.72, 2.83)  93.07% | ⨁◯◯◯ Very low |
| Balance/  TUG | 21 | RCT | Not serious^c^ | Very serious^d^ | Not serious | Not serious | 1254 | 1242 | −0.73 (−1.12, −0.34) 93.56% | ⨁⨁◯◯ Low |
| Balance/  FAB | 4 | RCT | Not serious^e^ | Serious^b^ | Not serious | Not serious | 108 | 169 | 2.80 (0.93, 4.66)  78.27% | ⨁⨁⨁◯ Moderate |
| Falls | 6 | RCT | Not serious^f^ | Serious^b^ | Not serious | Not serious | 696 | 695 | −0.30 (−0.54, −0.06)  71.77% | ⨁⨁⨁◯ Moderate |
| Overall | 43 | RCT | Serious^g^ | Serious^b^ | Not serious | Not serious | 2518 | 2766 |  | ⨁⨁◯◯ Low |
| MD: Mean Difference, CI: confidence interval, **I^2^**: Heterogeneity, BBS: Berg Balance Scale, FAB: Fullerton Advance Balance Scale; TUG: Time Up and Go, No: Number, RCT: Randomised Control Trial  **Explanations**  a. A low risk of bias is presented in 33% of studies. Some concern is presented in 44% of studies.  b. Presence of substantial to considerable heterogeneity in the trials.  c. A low risk of bias was reported in 71% of studies.  d. Presence of considerable heterogeneity in the trials.  e. A low risk of bias is presented in 66.7% of studies, and none presented a high risk of bias.  f. A low risk of bias is presented in 83% of the studies.  g. Overall, 58% of studies presented a low risk of bias. Some concern was found in 21% of the included studies.  A high risk of bias was presented in 21% of studies. | | | | | | | **GRADE Working Group grades of evidence** **High certainty:** we are very confident that the true effect lies close to that of the estimate of the effect. **Moderate certainty:** we are moderately confident in the effect estimate: the true effect is likely to be close to the estimate of the effect, but there is a possibility that it is substantially different. **Low certainty:** our confidence in the effect estimate is limited: the true effect may be substantially different from the estimate of the effect. **Very low certainty:** we have very little confidence in the effect estimate: the true effect is likely to be substantially different from the estimate of effect. | | | |

**Supplementary file 4**: Contour-enhanced funnel plots illustrating effect size (1, 5, 10%) in studies of dual-task exercise for balance improvement and fall reduction

BBS

TUG
